# Longitudinal Associations and Gender-Stratified Patterns in Adolescents’ Emotional and Behavioral Problems—Based on a Cross-Lagged Panel Network Model

**DOI:** 10.3390/bs16101761

**Published:** 2026-09-28

**Authors:** Yuanchao Hu, Fangfang Ding, Tongshuang Yuan, Kai Liu, Yujie Cui, Liqiang Zhang, Chaofan Zhang, Chengbin Zheng, Yaning Su, Liru Pan, Songli Mei

**Affiliations:** School of Public Health, Jilin University, Changchun 130021, China

**Keywords:** emotional and behavioral problems, adolescents, cross-lagged panel network analysis, gender-stratified patterns

## Abstract

Adolescence is a critical period characterized by heightened emotional and behavioral challenges, profoundly impacting young people’s social adaptation and future development. Previous research has predominantly employed traditional latent variable analysis to examine factors associated with emotional and behavioral problems, with less attention to the underlying symptom-level structure and prospective associations of these problems. This study utilized longitudinal data from the China Family Panel Studies (CFPS) collected in 2018 (T1) and 2022 (T2). It combined contemporaneous network analysis with cross-lagged panel network analysis (CLPN), employing the 14-item adolescent problem behavior measure included in the CFPS to examine contemporaneous and prospective symptom-level associations among emotional and behavioral problems in adolescents and to compare gender-stratified networks. The study included 1082 adolescents (mean age at T1 = 12.09 ± 1.54), with boys accounting for 54.5%. In the contemporaneous networks, worry about finishing homework (I10) had the highest expected influence at both waves, although other highly connected nodes differed across networks. In the CLPN, worry about having someone to play with at school (I11) and sadness (I7) had the highest out-expected influence (O-EI) values, whereas difficulty paying attention (E4) and being easily distracted (E6) had the highest in-expected influence (I-EI) values. Permutation tests did not provide evidence of significant between-gender differences. The findings provide a symptom-level perspective on contemporaneous and prospective associations among adolescent emotional and behavioral problems and identify symptom areas for further longitudinal and intervention research.

## 1. Introduction

Adolescence is a crucial transitional phase from childhood to adulthood, marked by significant changes in physiology, psychology, and cognition that influence thinking patterns and decision-making behaviors (Koenig et al., 2025). However, precisely during this critical period, adolescents’ emotional regulation abilities are not fully developed (Hatzenbuehler et al., 2008). When confronted with pressures from school, family, and society (Viner et al., 2012), they experience heightened levels of negative emotions and exhibit stronger emotional responses (Van Beveren et al., 2019), making them more susceptible to developing emotional and behavioral problems (EBPs) (Paus et al., 2008). The World Health Organization (2025) notes that depression, anxiety disorders, and behavioral disorders are major contributors to illness and disability among adolescents. A study from China indicated that the prevalence of emotional and behavioral problems among children and adolescents aged 6 to 16 was 17.6%. Compared with younger age groups, the prevalence was higher in older age groups (Y. Cui et al., 2021). This not only has a series of negative impacts on adolescents’ academic performance and social adaptation but also increases the risk of developing mental disorders in adulthood (Mulraney et al., 2021; Offerman et al., 2025; Wang et al., 2016). Previous studies have largely adopted a traditional perspective of analyzing relationships between variables, with less attention to the symptom-level interrelationships among adolescents’ emotional and behavioral problems. Consequently, this study aims to examine adolescents’ emotional and behavioral problems from a symptom-level network perspective, and to explore their contemporaneous network structures and prospective associations, thereby informing further longitudinal and intervention research.

### 1.1. Emotional and Behavioral Problems in Adolescents

Emotional and behavioral problems in adolescents are commonly described in terms of internalizing and externalizing problems. Internalizing problems are characterized by emotional disturbances such as anxiety and depression, while externalizing problems are marked by disruptive behaviors like conduct disorder and attention-deficit/hyperactivity disorder (ADHD) (Ogundele, 2018).

A study of Turkish adolescents found that psychological abuse predicts emotional and behavioral problems through the mediating effects of psychological resilience and self-esteem (Arslan, 2016). Another study of 10,437 adolescents across six urban areas worldwide found that childhood adversity and peer bullying are both associated with emotional and behavioral problems (Fine et al., 2022). Traditional research often treats emotional and behavioral problems as a single variable, driven by underlying psychological traits or various disorder factors (Black et al., 2022). This approach overlooks the interrelationships between different symptoms and their potential effects under mutual interaction. The network theory of mental disorders offers a new perspective to address this issue (Borsboom, 2017). This theory posits that mental disorders arise from the interactions between symptoms, rather than being caused by underlying diseases. Within this framework, mental disorders are viewed as interconnected networks, where symptoms are considered “nodes” and the interactions between symptoms form the “edges” connecting these nodes (Borsboom & Cramer, 2013). Researchers identify centrality indicators by analyzing the network structure formed by various symptom indicators, thereby pinpointing those indicators with the greatest influence on the onset, progression, and maintenance of disorders (Robinaugh et al., 2016). Recent studies have applied network analysis to internalizing problems (e.g., anxiety and depression) in adolescents. For instance, a study examining the association between depression and loneliness among 311 adolescents in Polish juvenile detention centers revealed that loneliness correlated with four affective depressive symptoms, which in turn influenced somatic symptoms (Grygiel & Dąbrowska, 2025). Song et al. (2025) conducted a cross-sectional network analysis on depressive symptoms among adolescents from different educational stages in China, finding that negative emotions and fatigue were core symptoms permeating the entire adolescent depression network.

Previous studies have suggested longitudinal associations between internalizing and externalizing problems. For instance, adolescents with depressive symptoms may be rejected by peers. To avoid isolation, they might choose to join deviant peer groups (Brendgen et al., 2000)—groups composed of individuals exhibiting antisocial, rule-breaking, or maladaptive behaviors—thereby increasing the likelihood of externalizing behaviors (Keskin et al., 2023). Conversely, the family conflicts or damaged interpersonal relationships associated with externalizing problems may further exacerbate internalizing symptoms (Capaldi & Stoolmiller, 1999), thus creating a vicious cycle between internalizing and externalizing issues. This interaction suggests that when studying adolescent mental health, it is essential to simultaneously examine the interplay between internalizing and externalizing problems to better understand patterns of symptom development.

Sun et al. (2025) utilized cross-sectional data to assess gender differences in the network structures of behavioral and emotional problems among Chinese children and adolescents. The results reveal that anxiety, depression, and aggressive behavior constitute common core issues in both boys’ and girls’ network structures. Compared with children, adolescents exhibit more pronounced behavioral and emotional problems. Chen et al. (2023) indicated that emotional problems adversely affect adolescents and may further be associated with suicidal thoughts. However, most current studies using network analysis to explore adolescent emotional and behavioral problems rely on cross-sectional data, which only reveal static relationships between symptoms at a single point in time and fail to capture temporal and directional relationships between symptoms. In reality, adolescents’ emotional and behavioral problems may change over time with changes in developmental environments and societal contexts. Consequently, core nodes identified at a single time point only indicate their relative importance within the cross-sectional network. They cannot characterize prospective directional associations between symptoms across time (Bos et al., 2017; S. Liu et al., 2022). Based on this, the cross-lagged panel network (CLPN) model was introduced by Wysocki et al. (2025). This model integrates cross-lagged panel analysis into network research, constructing a directed network of symptom relationships. It incorporates both autoregressive effects and cross-lagged effects, allowing for the examination of prospective directional associations between symptoms across measurement occasions. Furthermore, by calculating the in-expected influence (I-EI) and the out-expected influence (O-EI), the model characterizes the relative centrality of symptoms based on their incoming and outgoing prospective associations (Funkhouser et al., 2021).

### 1.2. Gender-Related Patterns

Previous studies have reported gender-related differences in the manifestation of emotional and behavioral problems among adolescents. The social role theory suggests that sociocultural expectations and societal norms may shape individuals’ behavioral expressions and psychological responses (Eagly, 1987). For instance, adolescent girls are more prone to internalizing emotional problems such as anxiety and depression. One study found that the prevalence of depressive symptoms reported by adolescent girls is twice that of boys (Girgus & Yang, 2015). Compared with boys, girls with depression more frequently exhibit symptoms like difficulty concentrating, increased appetite, weight gain, and sleep disturbances (Bennett et al., 2005), while boys tend to display behavioral issues such as aggression and hyperactivity (Matos et al., 2017). Although existing studies have shown that boys and girls differ in the expression of internalizing and externalizing problems, most research primarily relies on cross-sectional data (Luo et al., 2019; Sun et al., 2025), and potential gender-related variation in the prospective symptom-level associations of emotional and behavioral problems remains insufficiently understood. Therefore, examining potential gender-related patterns may provide a more nuanced understanding of adolescent emotional and behavioral problems.

### 1.3. The Current Study

In summary, adolescence is a critical stage during which emotional and behavioral problems in young people often co-occur and are interconnected. It is therefore essential to examine these issues concurrently within a unified framework in order to gain a comprehensive understanding of the symptom-level interrelationships and prospective associations. Based on this, this study followed a cohort of participants aged 10–15 years at baseline (T1) over a four-year interval, with participants aged 14–19 years at follow-up (T2). Accordingly, the present study had three specific aims: (1) to characterize the contemporaneous networks of emotional and behavioral problems at T1 and T2; (2) to examine prospective symptom-level associations from T1 to T2 using the CLPN; and (3) to examine potential gender-related variation in the longitudinal network patterns.

## 2. Methods

### 2.1. Participants

This study utilized data from the CFPS, a nationwide, comprehensive longitudinal social survey conducted by the Institute of Social Science Survey (ISSS) at Peking University. The CFPS collects data on China’s social, economic, demographic, educational, and health aspects, encompassing information at community, household, and individual levels (Xie & Lu, 2015). The project received approval from the Peking University Ethics Committee (Approval No. IRB00001052-14010).

Participants eligible for the present study were aged 10–15 years at baseline (T1). Of 2607 eligible participants, 1516 were matched to the 2022 wave. After applying the T2 age, covariate, and emotional and behavioral problems (EBPs) data criteria, the final analytic sample comprised 1082 participants from 966 households, including 112 households with more than one adolescent. Participants had a mean age of 12.09 years (SD = 1.54) at T1 and 16.08 years (SD = 1.54) at T2, and all were enrolled in school at T2. The participant selection process, demographic characteristics, and T2 age distributions are presented in Supplementary Figure S1, Table 1, and Supplementary Table S1, respectively.

EBPs responses outside the valid range of 1–5 were treated as missing, and the primary analyses were based on complete cases with valid EBPs and covariate data at both waves. Missing T1 parental education information was supplemented with corresponding 2022 information when available. Missingness occurred predominantly at the questionnaire-block level (Supplementary Table S2). Participants included in the final analytic sample were younger at baseline than those excluded, whereas no significant differences were observed in gender, household registration status, or baseline internalizing and externalizing problem levels (Supplementary Table S3).

### 2.2. Measures

#### Emotional and Behavioral Problems

Adolescents’ EBPs were assessed using the 14-item adolescent problem behavior measure included in the CFPS. According to the User Guide for China Family Panel Studies 2018, the 14 items were adopted from the Early Childhood Longitudinal Study (ECLS) and comprise two domains: internalizing problem behaviors and externalizing problem behaviors (Wu et al., 2021). The internalizing domain consists of eight items, whereas the externalizing domain consists of six items. This set of internalizing and externalizing problem behavior items has also been used in previous research among Chinese children (Lu et al., 2018). Detailed descriptions of all 14 items are presented in Table 2.

All items were rated on a 5-point response scale ranging from 1 (“not at all true of me”) to 5 (“completely true of me”), with higher scores indicating greater levels of the corresponding problem behavior. In the network analyses, the 14 items were treated as separate nodes representing specific internalizing and externalizing problem behaviors. For concise presentation in the network figures, the original CFPS variable codes were abbreviated according to their domain and item number (e.g., QInt011 as I11 and QExt004 as E4).

Cronbach’s alpha was 0.665 at T1 and 0.742 at T2 for the internalizing domain, and 0.684 at T1 and 0.661 at T2 for the externalizing domain. For all 14 items combined, Cronbach’s alpha was 0.784 at T1 and 0.811 at T2. Item-level descriptive statistics at T1 and T2 and by gender are provided in Supplementary Tables S6 and S7.

### 2.3. Data Analysis

Descriptive statistics and correlation analyses were conducted using SPSS 24.0, and correlation heat maps of the 14 EBPs items at T1 and T2 are presented in Supplementary Figure S2. R 4.4.2 was used for the contemporaneous network and the CLPN analyses.

To preliminarily assess measurement comparability, separate ordinal confirmatory factor analyses (CFAs) were conducted for the CFPS-defined internalizing and externalizing domains at T1 and T2 using the Weighted Least Squares Mean and Variance Adjusted (WLSMV) estimation. Because the domain-specific one-factor models showed mixed to inadequate fit, hierarchical factorial invariance testing was not pursued. Instead, item-level descriptive patterns were examined across waves and between boys and girls as supplementary comparability diagnostics; results are provided in Supplementary Tables S5–S7.

Before contemporaneous network estimation, each EBPs item was regressed on age, gender, household registration status, paternal education, and maternal education, and the resulting residuals were used for network estimation. In addition, potential node redundancy was assessed separately at T1 and T2 using the Goldbricker procedure (Levinson et al., 2018). Node pairs were considered potentially redundant when fewer than 25% of their correlations with the remaining nodes differed significantly (*p* < 0.05) and their inter-item correlation was at least 0.50; dependent correlations with overlapping variables were compared using the Hittner et al. (2003) method. The results of the redundancy assessment are provided in Supplementary Table S4.

The five-category EBPs items were treated as approximately continuous in the primary analysis, as five-category ordinal variables can often be reasonably approximated as continuous (Rhemtulla et al., 2012), and a Gaussian graphical model (GGM) was estimated using estimateNetwork with default = “huge”, with network regularization performed using the graphical least absolute shrinkage and selection operator (LASSO) (Epskamp et al., 2018; Friedman et al., 2008). The extended Bayesian information criterion (EBIC) (γ = 0.50) was used to select the optimal network model, with this relatively conservative setting chosen to reduce the inclusion of potentially spurious edges (Foygel & Drton, 2010). Expected influence (EI) was selected as the centrality metric for the contemporaneous networks. EI retains the signs of positive and negative edge weights (Robinaugh et al., 2016). Furthermore, as a sensitivity analysis, the contemporaneous networks were re-estimated using polychoric/polyserial correlations and EBICglasso (γ = 0.50) to examine whether the findings were robust to treating the five-category EBPs items as ordinal rather than approximately continuous. The 14 EBPs items and the same covariates were jointly included in the correlation matrix, with age treated as continuous and the remaining variables as ordered; the 14-item EBPs subnetwork was then extracted from the full EBICglasso network.

The CLPN was estimated using the glmnet package (Friedman et al., 2010) and visualized with the qgraph package (Epskamp et al., 2012). The five-category EBPs items were likewise treated as approximately continuous in the CLPN. Age, gender, household registration status, paternal education, and maternal education at T1 were included as covariates. For each T2 item, a Gaussian LASSO regression (alpha = 1) was estimated using 10-fold cross-validation, with lambda.min selected as the penalty parameter and predictors internally standardized by glmnet. Covariates were entered according to their numeric coding (age as continuous, gender and household registration status as binary, and paternal and maternal education as ordinal) and penalized together with the symptom predictors; the gender-stratified models used the same estimation settings, with gender omitted as a covariate after stratification. Since the edges in the cross-lagged network model are directed, centrality indices were estimated to determine the importance of each variable. Variable centrality was assessed by estimating the I-EI and O-EI. I-EI represents the extent to which each node is predicted by other nodes, while O-EI indicates the extent to which each node predicts other nodes. For the CLPN centrality analyses, I-EI and O-EI were calculated from the original Gaussian regression coefficients (*b*) after setting the diagonal autoregressive/self-loop paths to zero; thus, only cross-lagged associations between different nodes contributed to these indices. CLPN edge weights were reported on the original Gaussian regression-coefficient scale (*b*), with positive and negative values indicating positive and negative prospective associations, respectively, and zero indicating no retained association. To further examine whether potential item-content overlap affected the temporal network findings, sensitivity analyses were conducted by re-estimating the CLPN after separately omitting potentially overlapping items. In addition, survey weights and household-level clustering were not incorporated into the network analyses.

Subsequently, the bootnet package was employed to assess edge-weight accuracy and centrality stability (Epskamp et al., 2018). Edge-weight accuracy was assessed using 1000 nonparametric bootstrap samples and corresponding 95% confidence intervals. For each bootstrap sample, the complete CLPN estimation procedure was rerun using the same model specification as in the original sample. Centrality stability was assessed using case-dropping bootstrap analyses and quantified by the correlation stability (CS) coefficient. Bootstrap difference tests were also used to compare node centrality estimates within each network. The CS coefficient ranges from 0 to 1, with values >0.25 indicating moderate stability and > 0.50 indicating strong stability (Epskamp et al., 2018).

Additionally, CLPNs were estimated separately for boys and girls. Between-gender differences were directly tested using a permutation-based comparison procedure adapted to the directed CLPN setting (van Borkulo et al., 2023), with 1000 label permutations while preserving the observed group sizes. At each permutation, the complete gender-specific CLPNs were re-estimated using the same model specification. Global comparisons examined the maximum absolute edge difference and the difference in overall absolute connectivity. For individual directed non-autoregressive edges, two-sided permutation *p*-values were calculated, with Benjamini-Hochberg false discovery rate (BH-FDR) and max-T family-wise error rate (FWER) corrections applied for multiple testing.

## 3. Results

### 3.1. Contemporaneous Networks

Figure 1 and Figure 2 present the covariate-adjusted contemporaneous network structures at T1 (2018) and T2 (2022), while Figure 3 presents the corresponding EI estimates for both networks. At T1, the three nodes with the highest EI were I10 (“I worry about finishing my homework”), E8 (“It is hard for me to finish my school work”), and E2 (“I often argue with other kids”). At T2, the three nodes with the highest EI were I10 (“I worry about finishing my homework”), I9 (“I worry about doing well in school”), and I5 (“I often feel lonely”).

Bootstrap 95% confidence intervals for edge weights are presented in Supplementary Figures S3 and S6. The CS coefficients for EI were 0.672 at T1 and 0.750 at T2, indicating strong centrality stability (Supplementary Figures S4 and S7). Bootstrap difference tests of node EI are presented in Supplementary Figures S5 and S8. As a further robustness check, the contemporaneous networks were re-estimated by treating the five-category EBPs items as ordinal. The resulting network patterns were highly similar to those from the primary analyses, with signed edge-weight correlations of 0.975 at T1 and 0.972 at T2 and EI Spearman correlations of 0.916 and 0.934, respectively.

### 3.2. Temporal Network

Figure 4 displays the cross-lagged panel network of adolescents’ emotional and behavioral problems after adjustment for the covariates, with autoregressive paths omitted. The corresponding network including autoregressive paths is presented in Supplementary Figure S9. The largest cross-lagged coefficients were observed for I11 (“I worry about having someone to play with at school”) → I10 (“I worry about finishing my homework”) (*b* = 0.104), E6 (“I get distracted easily”) → E4 (“It is hard for me to pay attention”) (*b* = 0.101), and I11 (“I worry about having someone to play with at school”) → I9 (“I worry about doing well in school”) (*b* = 0.097). The centrality indices of nodes are shown in Figure 5. The two nodes with the highest O-EI values were I11 (“I worry about having someone to play with at school”) and I7 (“I feel sad a lot of the time”), while E4 (“It is hard for me to pay attention”) and E6 (“I get distracted easily”) had the highest I-EI values. Bootstrap 95% confidence intervals for edge weights are presented in Supplementary Figure S10, with numerical estimates and corresponding confidence intervals for the focal cross-lagged paths reported in Supplementary Table S8. The correlation stability coefficients were 0.284 for O-EI and 0.361 for I-EI, indicating moderate stability (see Supplementary Figure S11). Bootstrap difference tests of O-EI and I-EI are presented in Supplementary Figure S12. Sensitivity analyses yielded generally consistent centrality patterns and major longitudinal findings across the alternative item configurations (see Supplementary Table S4).

### 3.3. Gender-Stratified Networks

Figure 6 displays the gender-specific cross-lagged panel networks with autoregressive paths omitted. The corresponding networks including autoregressive paths are presented in Supplementary Figure S13. Direct permutation-based comparisons did not provide evidence of significant between-gender differences in the maximum absolute edge difference (*p* = 0.171) or global absolute connectivity (*p* = 0.221). Moreover, no individual directed edge difference remained statistically significant after BH-FDR or max-T correction (Supplementary Table S10).

Descriptively, in the girls’ network, the largest-magnitude cross-lagged coefficient was observed for I11 (“I worry about having someone to play with at school”) → E4 (“It is hard for me to pay attention”) (*b* = 0.137). In the boys’ network, the largest-magnitude coefficient was observed for E2 (“I often argue with other kids”) → E4 (*b* = −0.110), whereas the largest positive coefficient was observed for I5 (“I often feel lonely”) → E4 (*b* = 0.104). Numerical estimates and bootstrap confidence intervals for the focal gender-specific cross-lagged paths are reported in Supplementary Table S9.

Figure 7 presents the centrality estimates for the boys’ and girls’ networks. Descriptively, in the boys’ network, node I7 (“I feel sad a lot of the time”) exhibited the largest O-EI, while node E4 (“It is hard for me to pay attention”) showed the largest I-EI. In the girls’ network, node I11 (“I worry about having someone to play with at school”) had the largest O-EI, and node E4 (“It is hard for me to pay attention”) demonstrated the largest I-EI. Bootstrap 95% confidence intervals for gender-specific edge weights are presented in Supplementary Figure S14. The CS coefficients were 0.283 for both O-EI and I-EI in boys, 0.283 for O-EI in girls, and 0.128 for I-EI in girls, indicating moderate stability for the former three indices but low stability for girls’ I-EI (Supplementary Figure S15). Bootstrap difference tests of O-EI and I-EI within the girls’ and boys’ networks are presented in Supplementary Figures S16 and S17.

## 4. Discussion

Although a significant correlation between adolescent emotional and behavioral problems has been demonstrated (Oh et al., 2020), little is known about how these problems are interconnected and prospectively associated at the symptom level. Therefore, grounded in psychopathology theory and employing a longitudinal design, this study built both contemporaneous and cross-lagged panel networks to examine symptom-level associations among emotional and behavioral problems in Chinese adolescents. In the contemporaneous networks, I10 (“I worry about finishing my homework”) had the highest EI at both T1 and T2. In the temporal network, I11 (“I worry about having someone to play with at school”) and I7 (“I feel sad a lot of the time”) had the two highest O-EI values, whereas E4 (“It is hard for me to pay attention”) and E6 (“I get distracted easily”) had the two highest I-EI values. In addition, although the gender-stratified networks showed descriptively different patterns, direct permutation tests did not provide evidence of statistically significant between-gender differences. From a dimensional psychopathology perspective, these findings highlight symptom-level heterogeneity within internalizing and externalizing domains (Kotov et al., 2017).

### 4.1. Contemporaneous Networks of EBPs

At T1 and T2, the nodes with higher EI were not entirely the same. At T1, the nodes I10 (“I worry about finishing my homework”) and E8 (“It is hard for me to finish my school work”) demonstrated relatively high EI. This finding may be closely related to the educational context faced by Chinese adolescents. Academic demands often increase during adolescence, accompanied by more frequent examinations and heightened expectations regarding academic achievement (Huang et al., 2026). Moreover, academic success is often highly valued by both parents and schools, making educational performance an important source of self-evaluation and social approval (Chen et al., 2021). Consequently, concerns about completing assignments and meeting academic requirements may have become particularly salient stressors during this developmental period.

Adolescence is a period characterized by substantial developmental changes in cognitive, emotional, and social functioning. Against this developmental background, adolescents may therefore experience greater difficulties adapting to increasing academic demands and performance expectations (Jiang et al., 2022; Zhou et al., 2025), making it harder for them to cope effectively with these pressures. This may contribute to diminished perceived control over their studies and an increased likelihood of experiencing anxiety and fear (Steare et al., 2023).

Additionally, E2 (“I often argue with other kids”) also showed relatively high EI at T1. According to ecological systems theory (Bronfenbrenner, 1979), individuals develop within multi-level social ecosystems. This theory emphasizes linking human development to specific environments, exploring the emergence and evolution of individual behavior from a systemic and holistic perspective. As adolescents grow and enter the socialization stage, the importance of peer relationships in their development gradually increases, with adolescents showing significantly heightened dependence on and identification with peer groups (Roach, 2018). Consequently, maintaining positive peer relationships is an important developmental task during adolescence. Difficulties in peer interactions may be associated with poorer emotional and behavioral adjustment among adolescents. One factor that may be associated with peer conflicts is social comparison, which becomes increasingly salient during adolescence.

During interpersonal interactions, individuals face strong social comparison pressures (Festinger, 1954), such as evaluating themselves against peers in academic performance, physical appearance, and social media popularity (J. Li et al., 2021; Nesi & Prinstein, 2015). Such comparisons may increase interpersonal sensitivity and competitiveness, thereby creating greater opportunities for disagreements and conflicts among peers. These conflicts may further undermine adolescents’ sense of belonging and social support, thereby increasing their vulnerability to emotional and behavioral problems.

At T2, I10 (“I worry about finishing my homework”) remained a relatively central node, while I9 (“I worry about doing well in school”) also showed relatively high centrality within the network. These findings may reflect the salience of academic pressure and emotional distress among adolescents at T2. During adolescence, driven by dual cognitive and social development, adolescents increasingly recognize the importance of personal responsibility, with academic advancement becoming a primary task (Pienyu et al., 2024). Confronted with selective examinations like the high school entrance exam and college entrance exam amid intensifying competition, adolescents may experience substantial academic pressure, which may be associated with persistent anxiety and self-doubt (Ye et al., 2025). Simultaneously, influenced by traditional cultural values (such as the concept of “face”), adolescents are highly sensitive to others’ evaluations of their academic performance, fearing negative judgments for underachieving (H. Liu et al., 2024). Furthermore, parental educational anxiety and high expectations may exacerbate adolescents’ psychological burdens (D. Li et al., 2024). Research indicates that when parents set overly ambitious goals, the gap between a child’s actual capabilities and parental expectations creates immense pressure (Kaynak et al., 2023), intensifying feelings of anxiety and fear.

Additionally, I5 (“I often feel lonely”) demonstrated relatively high EI, a finding consistent with the results of S. Liu et al. (2025). This finding may reflect the importance of social relationships during adolescence, as difficulties in peer relationships are often associated with feelings of social disconnection and isolation (Verity et al., 2025). Persistent loneliness may undermine adolescents’ sense of belonging and perceived social support, leading to negative cognitive biases, heightened emotional reactivity, and difficulties in emotion regulation (Jost et al., 2025). These psychological processes may subsequently increase the risk of anxiety, depressive symptoms, and other emotional and behavioral difficulties.

In summary, the contemporaneous networks at T1 and T2 showed complex patterns of associations among emotional and behavioral problems. School-related concerns, peer-related difficulties, and loneliness were among the relatively central features identified across the two estimated networks. These findings highlight the potential relevance of social relationships and emotional regulation, alongside academic support, for future intervention research.

### 4.2. Temporal Network of EBPs

The CLPN model indicated that the two symptoms with the highest O-EI were I11 (“I worry about having someone to play with at school”) and I7 (“I feel sad a lot of the time”). These findings suggested that I11 and I7 had relatively prominent outgoing prospective associations within the estimated network; however, these rankings should be interpreted cautiously given the moderate stability of O-EI (CS = 0.284).

These findings may be particularly relevant during adolescence, when peer relationships become increasingly important for social and emotional adjustment. As adolescence progresses, individuals gradually detach from the family environment, increasingly investing in social relationships beyond the nuclear family. Their need for social interaction intensifies (Lamblin et al., 2017), with peers becoming a vital source of support. Research indicates that close friendships positively correlate with emotional adaptation during adolescence (Zheng et al., 2024). According to self-determination theory, individuals’ psychological needs encompass autonomy, competence, and relatedness. The fulfillment of these needs is closely linked to mental health and behavioral outcomes (Deci & Ryan, 2000). When adolescents lack stable peer relationships, their need for relatedness remains unmet, making them susceptible to emotional issues like loneliness and sadness.

Further analysis of cross-lagged effects revealed that worry about having someone to play with at school (I11) showed prospective associations with other symptoms in the network. Paths with higher weights included I11 (“I worry about having someone to play with at school”) → I10 (“I worry about finishing my homework”) and I11 (“I worry about having someone to play with at school”) → I9 (“I worry about doing well in school”), suggesting that worry about having someone to play with at school was prospectively associated with higher subsequent worries about homework completion and school performance. According to the limited resources theory (Kahneman, 1973), individuals possess finite resources (such as time, energy, and attention). Persistent worry about having someone to play with at school may consume mental energy and attention, potentially reducing available resources for academic tasks and increasing vulnerability to academic worries. A study of Norwegian adolescents indicated that social anxiety during adolescence was associated with poorer high-school completion outcomes (Jystad et al., 2024). Consequently, future intervention research could examine approaches that focus on emotional regulation skills, positive peer relationships, and social skills. Such approaches may be relevant for addressing both interpersonal and academic difficulties.

### 4.3. Gender-Stratified Patterns

Although the permutation tests did not support statistically significant between-gender differences, some notable patterns were observed within the girls’ and boys’ networks.

In the girls’ network, I11 (“I worry about having someone to play with at school”) had the highest O-EI, and the largest-magnitude cross-lagged coefficient was observed for I11 → E4 (“It is hard for me to pay attention”) (*b* = 0.137). One possible interpretation is that concerns about peer relationships may be salient among adolescent girls, as previous research suggests that girls may place greater emphasis on maintaining social relationships and may be more sensitive to rejection and interpersonal difficulties (Bedrov & Gable, 2023; Rose & Rudolph, 2006). Persistent interpersonal concerns may also be related to later difficulties in attention. Previous research has suggested that interpersonal stress may be associated with negative cognitive styles and rumination, which may in turn be related to concentration difficulties (Hamilton et al., 2015).

In the boys’ network, I7 (“I feel sad a lot of the time”) had the highest O-EI, and a relatively large positive cross-lagged coefficient was observed for I7 → E13 (“I get in trouble for fighting with other kids”). One possible interpretation is that emotional distress may sometimes be accompanied by more externalizing forms of behavioral expression. Previous research has suggested that some boys may be less likely to express emotional distress directly and may instead display it through externalizing behaviors, a pattern that may be influenced in part by traditional gender-role expectations (R. F. Cui & Fiske, 2021; Hamilton et al., 2015).

Taken together, the findings observed in the girls’ and boys’ networks may provide useful hypotheses for future research, but should not be interpreted as evidence of established gender differences; further studies are needed to determine whether these findings are reproducible and reflect reliable gender-related variation.

### 4.4. Limitations and Future Directions

Several limitations should be considered. First, although several covariates were included, other contextual factors, such as family environment and social experiences, may still have influenced the findings. Reliance on adolescent self-report may also have introduced reporting bias. Future studies could incorporate broader contextual factors and multi-informant assessments.

Second, only two waves separated by four years were available. Thus, the CLPN captures prospective associations over a relatively long interval and cannot characterize shorter-term symptom dynamics or establish causal effects. Moreover, the estimated associations may partly reflect stable between-person differences rather than within-person symptom changes (Hamaker et al., 2015). The broad age ranges and the passage of time from 2018 to 2022 also limit the interpretation of T1–T2 differences as developmental changes.

Third, attrition and complete-case selection may have introduced selection bias. Household-level clustering was also not accounted for; dependence among adolescents from the same household may have affected the precision of the estimates. In addition, not incorporating the CFPS survey weights and complex sampling design may limit the population-level generalizability of the findings.

Fourth, measurement comparability across waves and gender groups could not be fully established. Some item-content overlap also cannot be completely ruled out, although the Goldbricker analysis identified no redundant node pairs and the sensitivity analyses yielded generally consistent findings.

Fifth, some temporal centrality estimates showed limited stability, particularly girls’ I-EI (CS = 0.128), and the corresponding node rankings should therefore be interpreted cautiously.

Finally, given the high proportion of participants with agricultural household registration and relatively low parental education levels, generalizability to more urban, socioeconomically advantaged, or clinical populations may be limited. Future studies with additional waves, shorter assessment intervals, improved measurement evaluation, and more diverse samples could further assess the robustness and generalizability of these findings.

## 5. Conclusions

This study examined contemporaneous and prospective symptom-level associations among emotional and behavioral problems in Chinese adolescents across two measurement occasions four years apart. The network analyses highlighted worries about having someone to play with at school, doing well in school, and finishing homework, together with sadness; worry about having someone to play with at school and sadness showed the highest O-EI in the temporal network. These findings provide a more detailed symptom-level understanding of emotional and behavioral problems and their prospective associations, and point to symptom areas that warrant further attention in longitudinal and intervention research. Gender-stratified analyses showed some notable patterns within the boys’ and girls’ networks, but permutation tests did not provide evidence of statistically significant between-gender differences; further research is needed to determine whether these patterns reflect reliable gender-related variation.

## Figures and Tables

**Figure 1:**
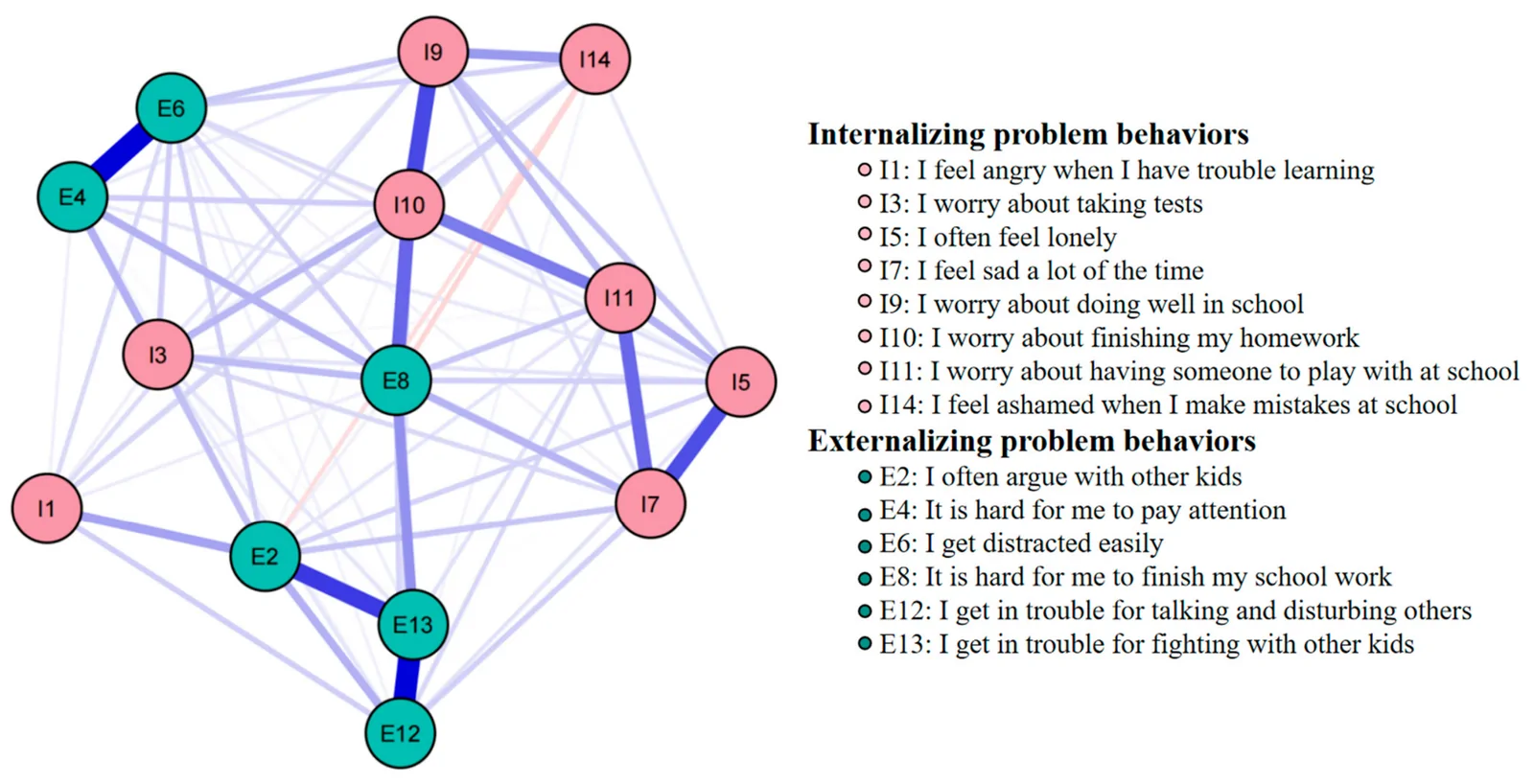
Contemporaneous network structures at T1. Note. Blue edges indicate positive regularized partial correlations, and red edges indicate negative regularized partial correlations. Thicker edges indicate stronger associations.

**Figure 2:**
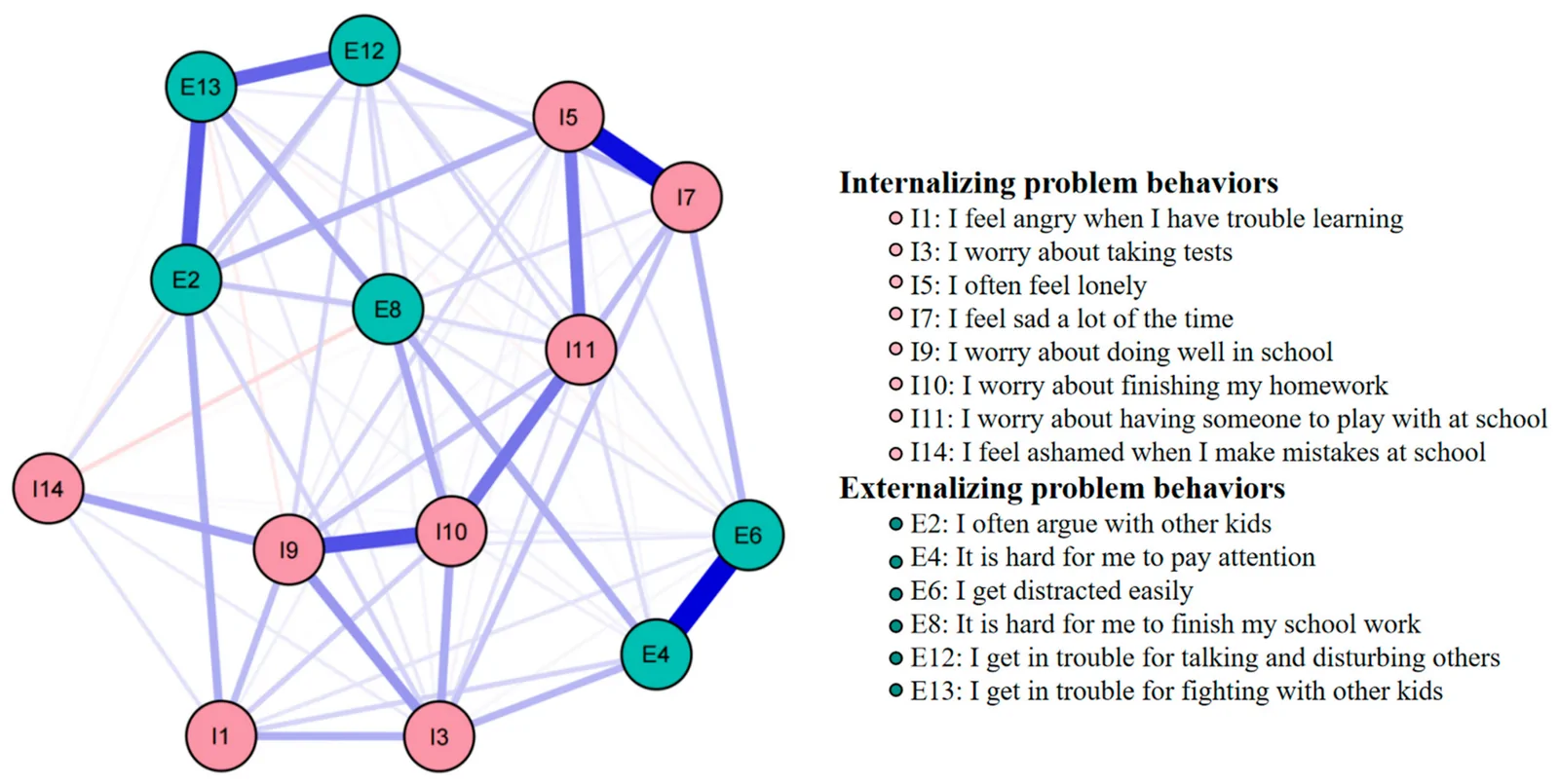
Contemporaneous network structures at T2. Note. Blue edges indicate positive regularized partial correlations, and red edges indicate negative regularized partial correlations. Thicker edges indicate stronger associations.

**Figure 3:**
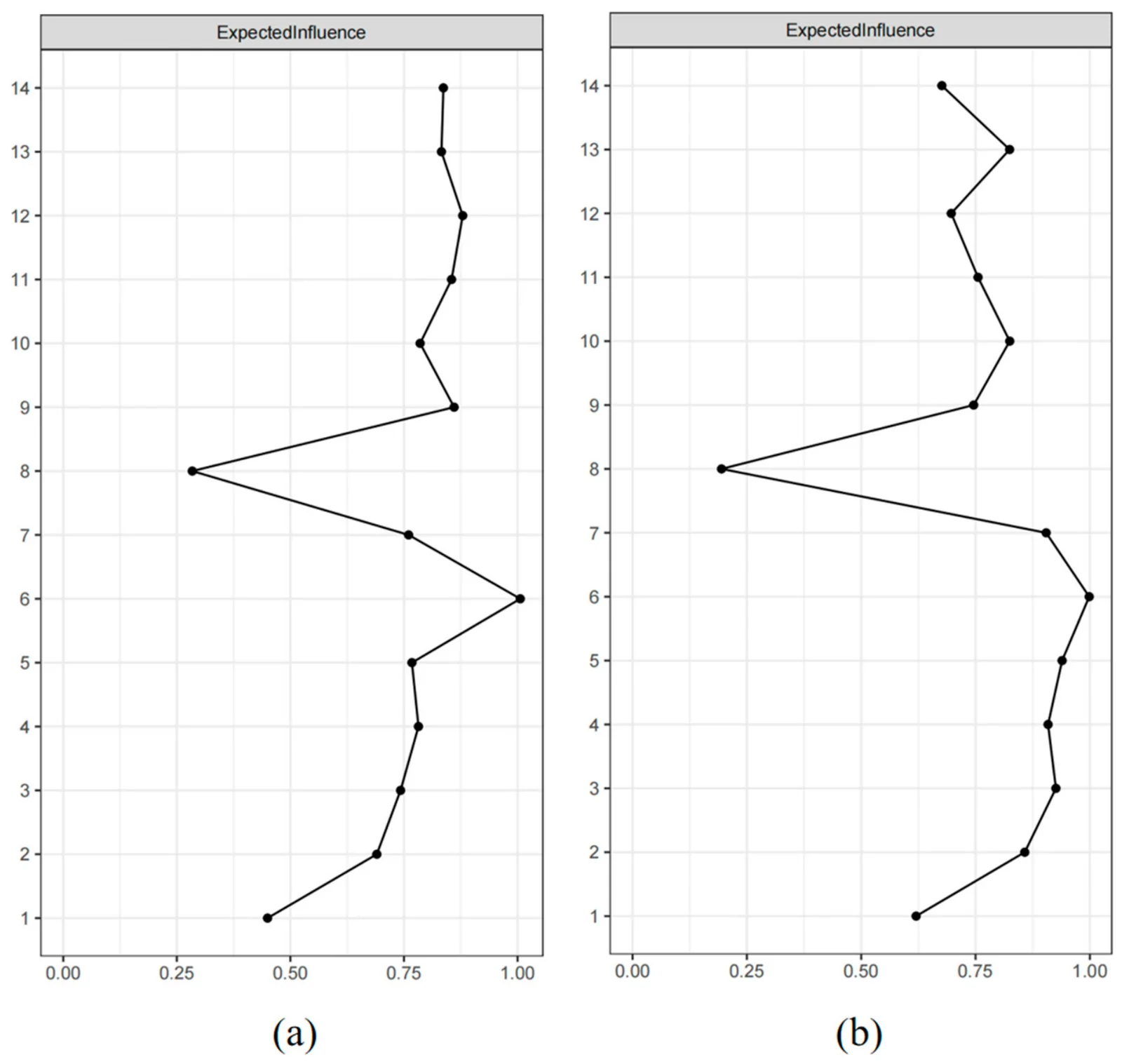
EI estimates for each node in the T1 and T2 contemporaneous networks. Note. Panels (**a**) and (**b**) present EI estimates for the T1 (2018) and T2 (2022) networks, respectively. Higher EI values indicate greater node centrality within the corresponding contemporaneous network.

**Figure 4:**
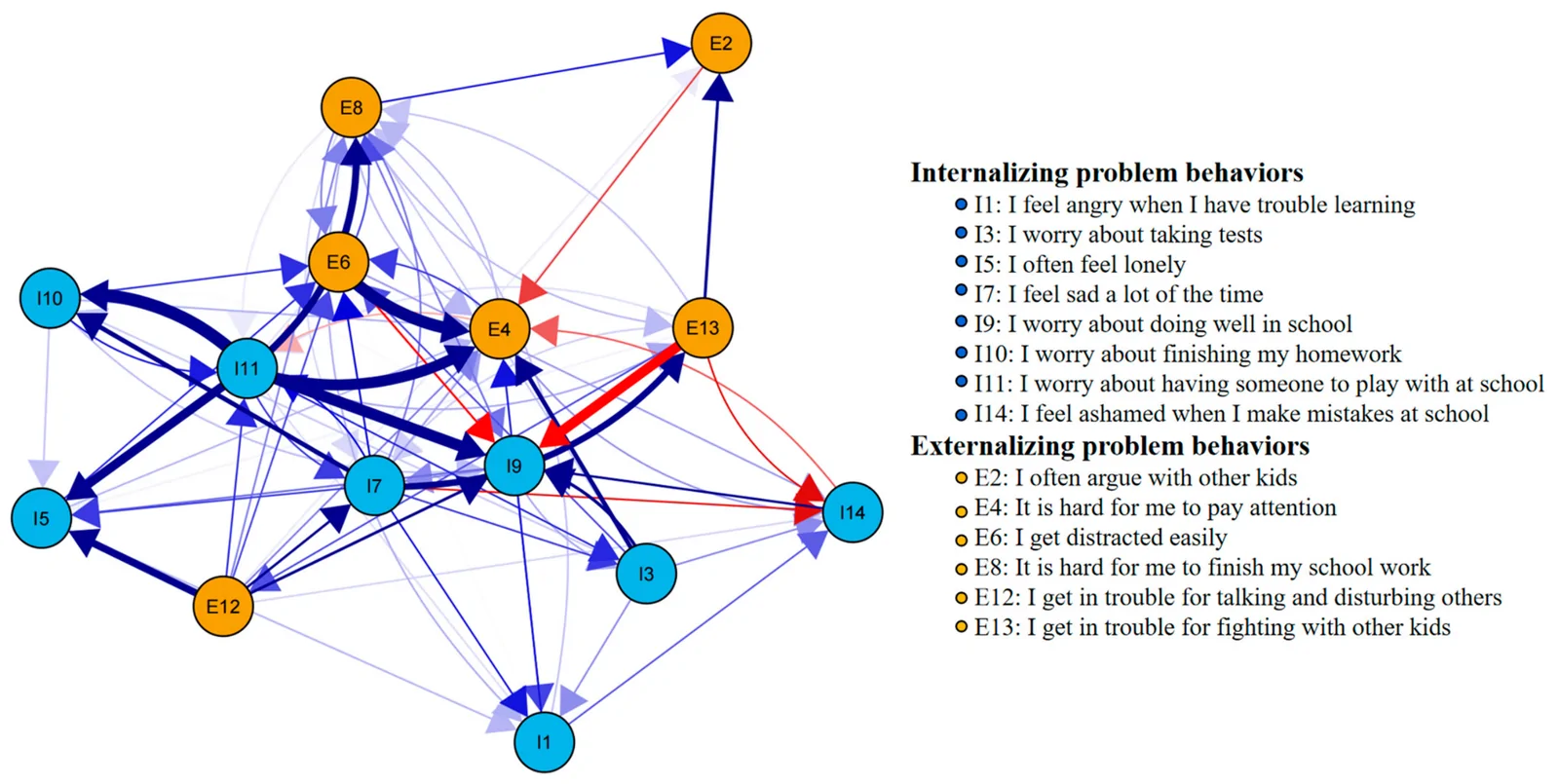
Cross-lagged panel network for T1 → T2 with autoregressive paths omitted. Note. Arrows indicate prospective associations from T1 to T2. Blue arrows indicate positive regression coefficients, whereas red arrows indicate negative regression coefficients. Arrows thickness represents the absolute magnitude of the Gaussian LASSO regression coefficient (*b*). Autoregressive paths are omitted.

**Figure 5:**
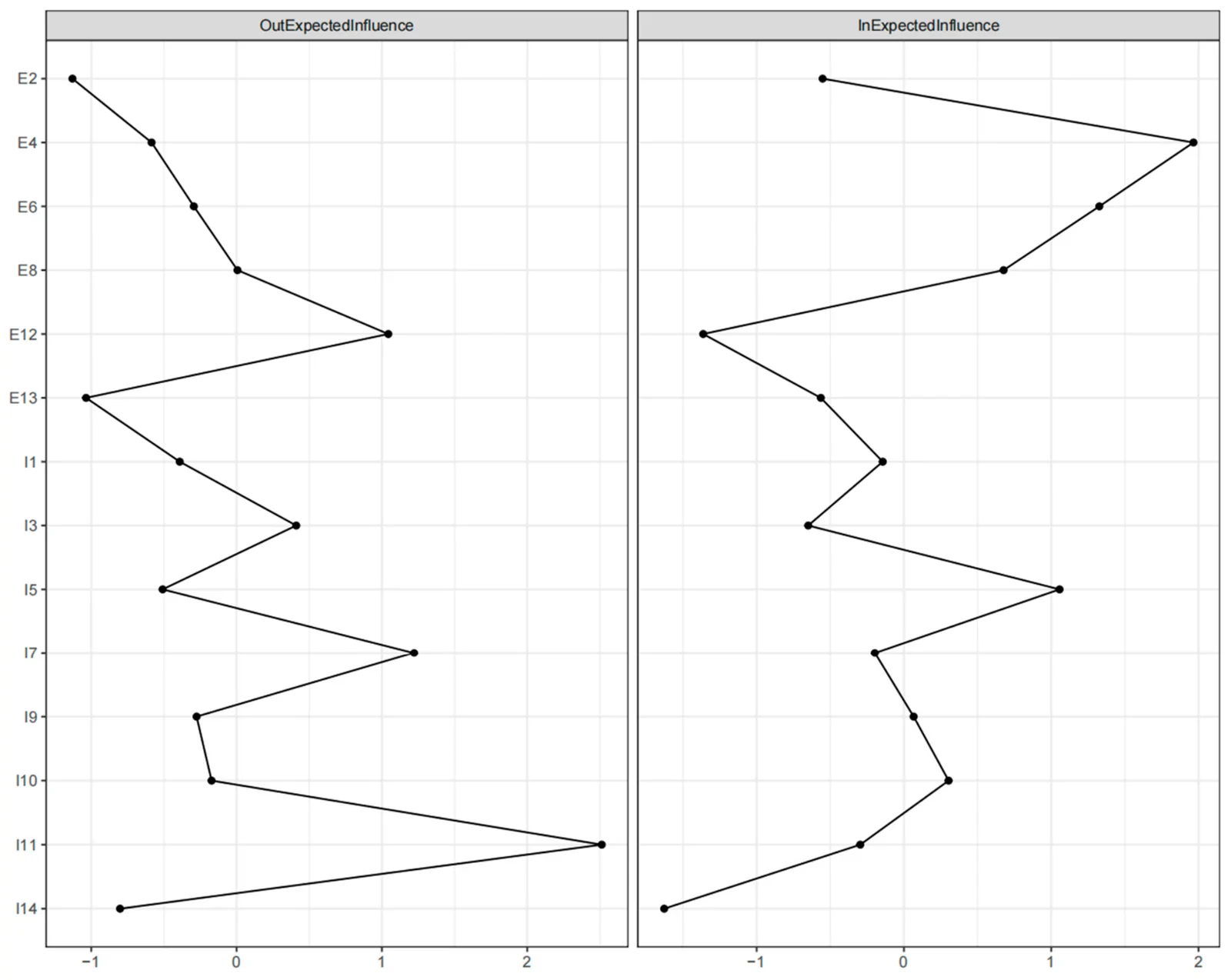
Centrality estimates in the T1 → T2 network. Note. I-EI and O-EI represent the extent to which each node is predicted by and predicts other nodes, respectively. Both were calculated from the original Gaussian regression coefficients (*b*), excluding autoregressive paths; values are shown as z-scores.

**Figure 6:**
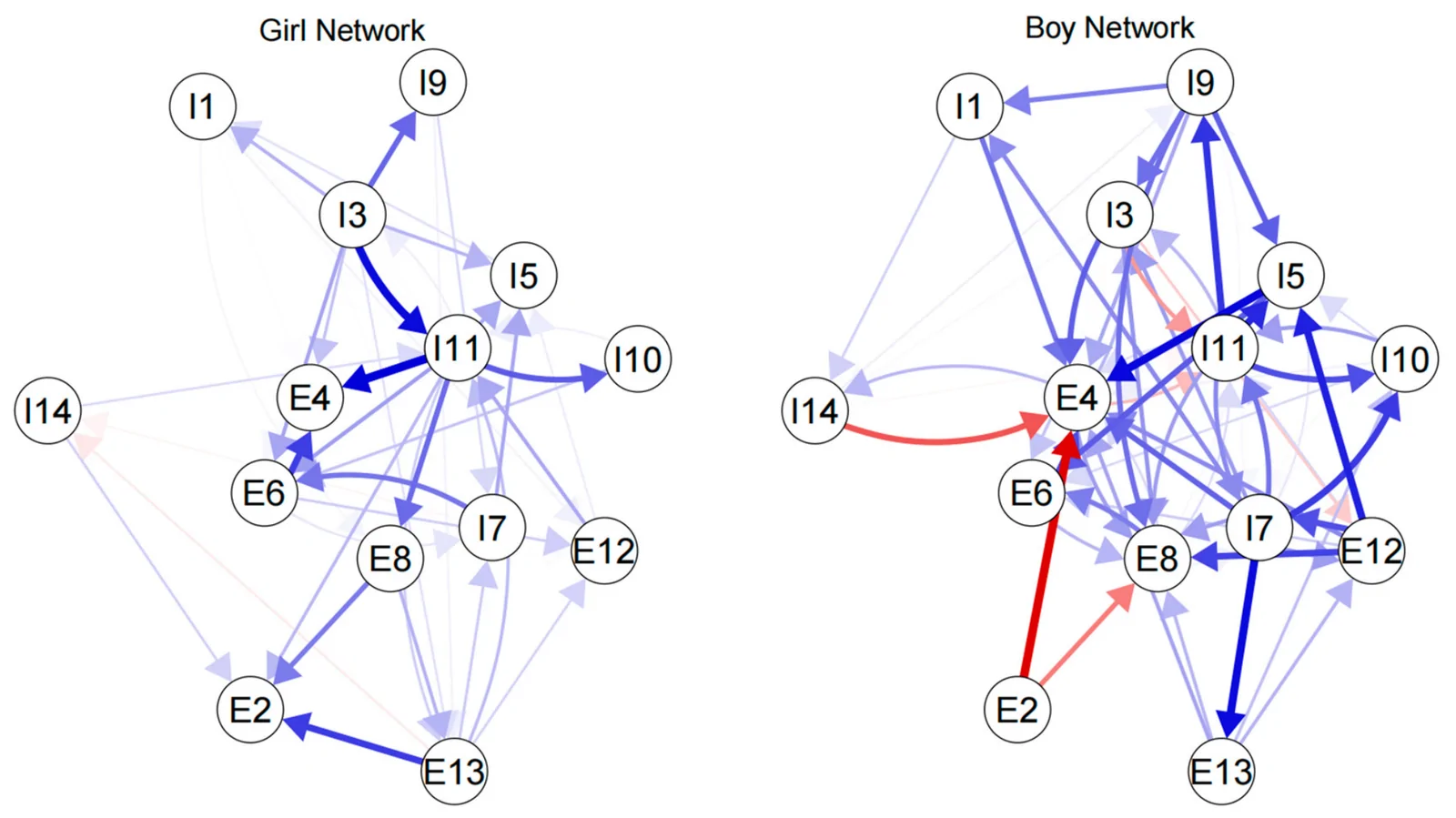
Cross-lagged panel network for adolescent Girls (**left**) and Boys (**right**). Note. Arrows indicate prospective associations from T1 to T2. Blue arrows indicate positive regression coefficients, whereas red arrows indicate negative regression coefficients. Arrows thickness represents the absolute magnitude of the Gaussian LASSO regression coefficient (*b*). I, internalizing items; E, externalizing items. See Table 2 for item descriptions. Autoregressive paths are omitted.

**Figure 7:**
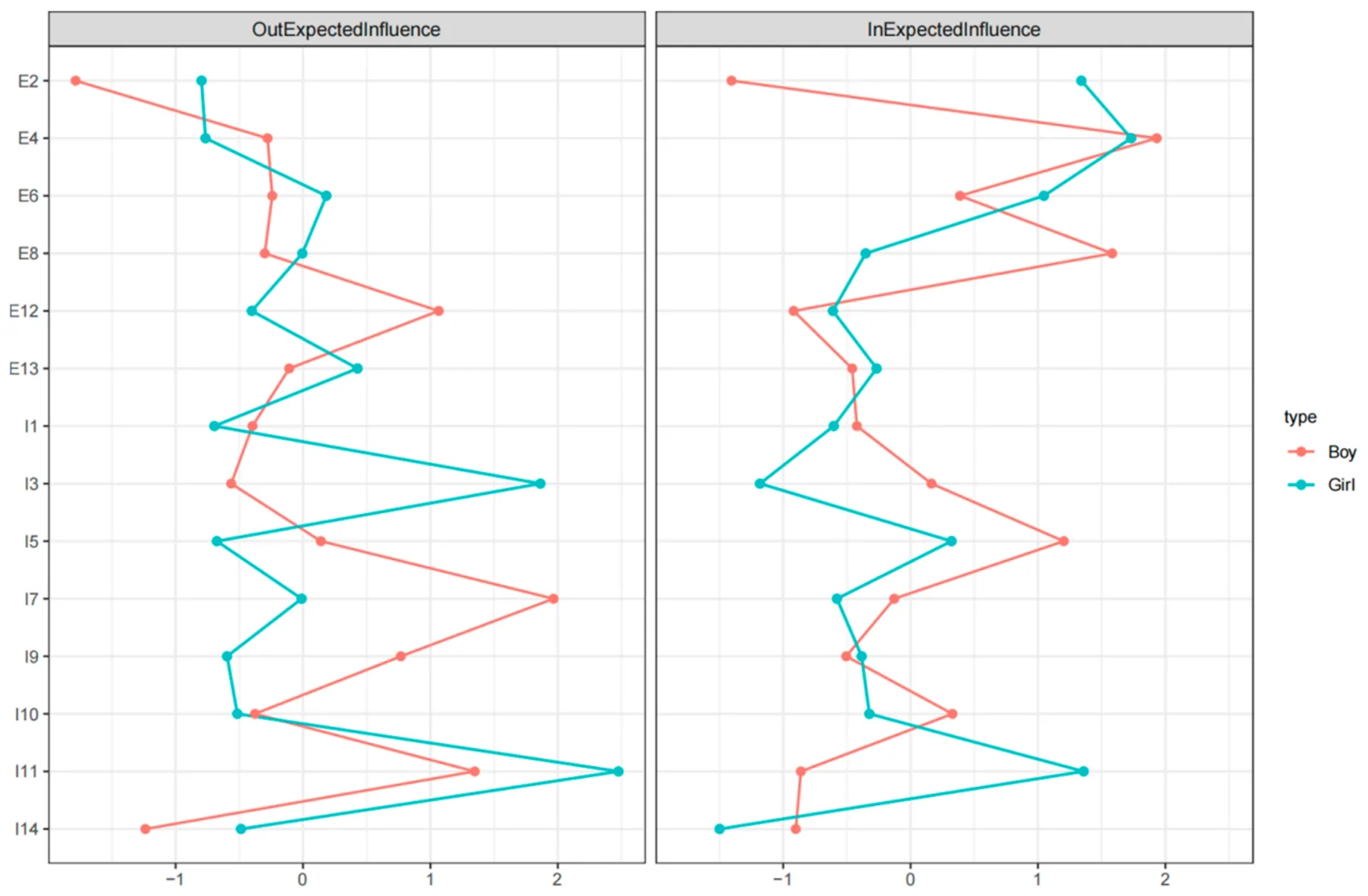
Standardized centrality estimates for girls and boys. Note. O-EI and I-EI were calculated from the original Gaussian regression coefficients (*b*), with autoregressive paths excluded. Centrality values are presented as z-scores for visualization.

**Table 1:** Demographic characteristics of the final analytic sample (*N* = 1082).

Variables		*n*	Percentage/*Mean* (*SD*)
Age at T1, years	10–15	1082	12.09 (1.54)
Age at T2, years	14–19	1082	16.08 (1.54)
Gender			
	Boys	590	54.5
	Girls	492	45.5
Household registration status			
	Agricultural	902	83.4
	Non-agricultural	180	16.6
Father’s educational level			
	Primary school and below	530	49.0
	Junior high school	368	34.0
	High school	128	11.8
	Associate’ s degree or higher	56	5.2
Mother’s educational level			
	Primary school and below	709	65.5
	Junior high school	274	25.3
	High school	59	5.5
	Associate’ s degree or higher	40	3.7

Note. Values are shown as *n* (%), and age is shown as *M* (*SD*).

**Table 2:** Item wording of the 14-item adolescent problem behavior measure.

Domain	Label	Item
Internalizing problem behaviors		
	I1	I feel angry when I have trouble learning
	I3	I worry about taking tests
	I5	I often feel lonely
	I7	I feel sad a lot of the time
	I9	I worry about doing well in school
	I10	I worry about finishing my homework
	I11	I worry about having someone to play with at school
	I14	I feel ashamed when I make mistakes at school
Externalizing problem behaviors		
	E2	I often argue with other kids
	E4	It is hard for me to pay attention
	E6	I get distracted easily
	E8	It is hard for me to finish my school work
	E12	I get in trouble for talking and disturbing others
	E13	I get in trouble for fighting with other kids

## Data Availability

The data used in this study are available through the China Family Panel Studies (CFPS) official data platform upon registration and approval. The data are not publicly available due to restrictions imposed by the CFPS Data User Agreement.

## Supplementary Materials

The following supporting information can be downloaded at: https://www.mdpi.com/article/10.3390/bs16101761/s1, Figure S1: Flowchart of participant selection; Figure S2: Correlation matrices of the 14 EBPs items at T1 and T2; Figure S3: Bootstrapped 95% CI of estimated edge weights in T1; Figure S4: EI stability of the network in T1; Figure S5: Bootstrapped test of node EI differences at T1; Figure S6: Bootstrapped 95% CI of estimated edge weights in T2; Figure S7: EI stability of the network in T2; Figure S8: Bootstrapped test of node EI differences at T2; Figure S9: Overall cross-lagged panel network including autoregressive paths; Figure S10: Bootstrap accuracy of edge-weight estimates in the overall cross-lagged panel network; Figure S11: Case-dropping bootstrap stability of O-EI and I-EI in the overall cross-lagged panel network; Figure S12: Bootstrap difference tests of O-EI and I-EI centrality in the overall cross-lagged panel network; Figure S13: Cross-lagged panel networks for adolescent girls (left) and boys (right), including autoregressive and cross-lagged paths; Figure S14: Bootstrap accuracy of edge-weight estimates in the gender-specific cross-lagged panel networks; Figure S15: Case-dropping bootstrap stability of O-EI and I-EI in the gender-specific cross-lagged panel networks; Figure S16: Bootstrap difference tests of O-EI and I-EI centrality in the girls’ cross-lagged panel network; Figure S17: Bootstrap difference tests of O-EI and I-EI centrality in the boys’ cross-lagged panel network; Table S1: Age distributions of the final analytic sample at T2 (*N* = 1082); Table S2: Missing and invalid responses to EBPs items among participants successfully matched to T2 and meeting the age criteria at both waves (*N* = 1513); Table S3: Baseline characteristics of participants included in and excluded from the final analytic sample; Table S4: Node-redundancy assessment and CLPN sensitivity analyses for potentially overlapping items; Table S5: Preliminary domain-specific ordinal CFA model fit used to assess suitability for factorial measurement-invariance testing; Table S6: Item-level descriptive statistics and across-wave distributional differences at T1 and T2; Table S7: Item-level descriptive statistics and gender-related distributional differences at T1 and T2; Table S8: Numerical results of the overall cross-lagged panel network and bootstrap confidence intervals for focal cross-lagged paths; Table S9: Numerical results of the gender-specific cross-lagged panel networks and bootstrap confidence intervals for focal cross-lagged paths; Table S10: Direct permutation-based comparisons of the girls’ and boys’ cross-lagged panel networks.
