# Supplementary material for: Longitudinal Associations and Gender-Stratified Patterns in Adolescents’ Emotional and Behavioral Problems—Based on a Cross-Lagged Panel Network Model
Source: Behav Sci (Basel). 2026 Sep 28;16(10):1761. doi: 10.3390/bs16101761 (PMC13701318; doi:10.3390/bs16101761)
Supplement: Supplementary file 1 [file behavsci-16-01761-s001.zip › behavsci-4489771-supplementary.pdf]

Longitudinal Associations and Gender-Stratified Patterns in Adolescents' Emotional and Behavioral Problems—Based on a Cross-Lagged Panel Network Model

Supplementary materials

Figure S1. Flowchart of participant selection

Figure S2. Correlation matrices of the 14 EBPs items at T1 and T2

Figure S3. Bootstrapped 95% CI of estimated edge weights in T1

Figure S4. EI stability of the network in T1

Figure S5. Bootstrapped test of node EI differences at T1

Figure S6. Bootstrapped 95% CI of estimated edge weights in T2

Figure S7. EI stability of the network in T2

Figure S8. Bootstrapped test of node EI differences at T2

Figure S9. Overall cross-lagged panel network including autoregressive paths

Figure S10. Bootstrap accuracy of edge-weight estimates in the overall cross-lagged panel network

Figure S11. Case-dropping bootstrap stability of O-EI and I-EI in the overall cross-lagged panel network

Figure S12. Bootstrap difference tests of O-EI and I-EI centrality in the overall cross-lagged panel network

Figure S13. Cross-lagged panel networks for adolescent girls (left) and boys (right), including autoregressive and cross-lagged paths

Figure S14. Bootstrap accuracy of edge-weight estimates in the gender-specific cross-lagged panel networks

Figure S15. Case-dropping bootstrap stability of O-EI and I-EI in the gender-specific cross-lagged panel networks

Figure S16. Bootstrap difference tests of O-EI and I-EI centrality in the girls' cross-lagged panel network

Figure S17. Bootstrap difference tests of O-EI and I-EI centrality in the boys' cross-lagged panel network

Table S1. Age distributions of the final analytic sample at T2 (N = 1,082)

Table S2. Missing and invalid responses to EBPs items among participants successfully matched to T2 and meeting the age criteria at both waves (N = 1,513)

Table S3. Baseline characteristics of participants included in and excluded from the final analytic sample

Table S4. Node-redundancy assessment and CLPN sensitivity analyses for potentially overlapping items

Table S5. Preliminary domain-specific ordinal CFA model fit used to assess suitability for factorial measurement-invariance testing

Table S6. Item-level descriptive statistics and across-wave distributional differences at T1 and T2

Table S7. Item-level descriptive statistics and gender-related distributional differences at T1 and T2

Table S8. Numerical results of the overall cross-lagged panel network and bootstrap confidence intervals for focal cross-lagged paths

Table S9. Numerical results of the gender-specific cross-lagged panel networks and bootstrap confidence intervals for focal cross-lagged paths

Table S10. Direct permutation-based comparisons of the girls' and boys' cross-lagged panel networks

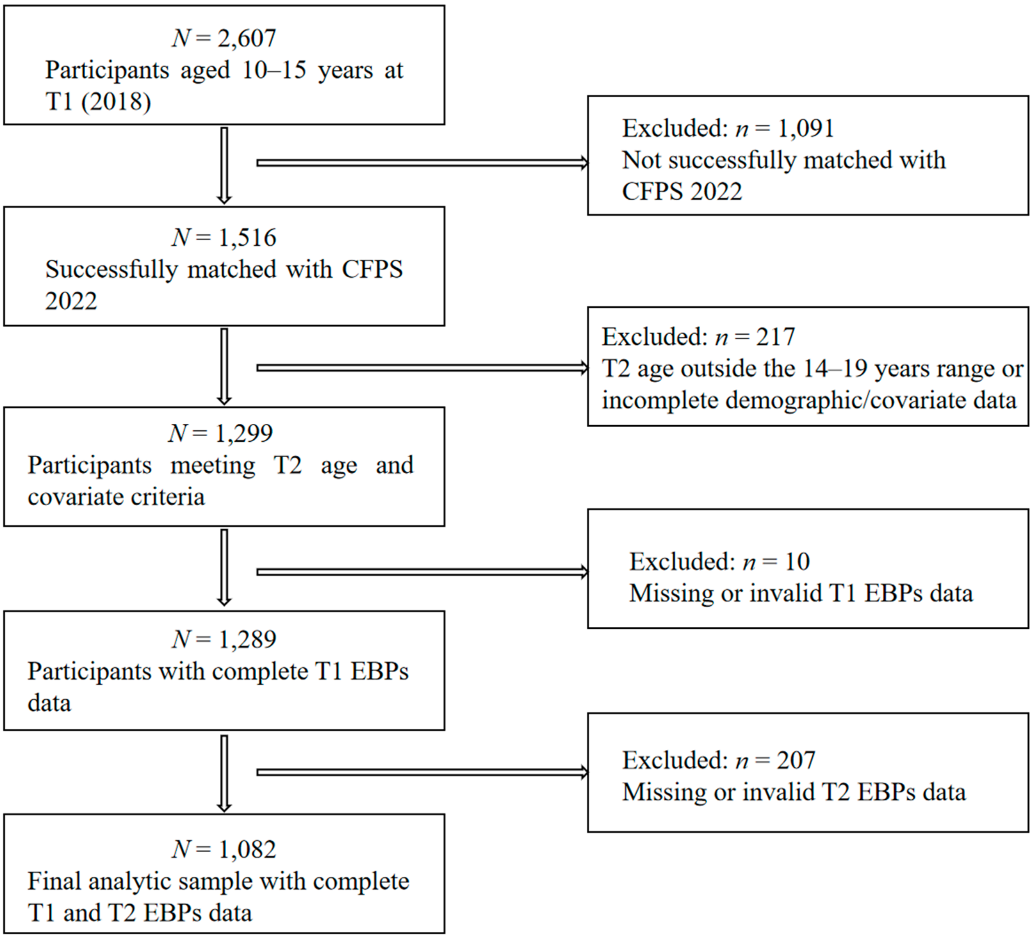

Figure S1. Flowchart of participant selection

(a)

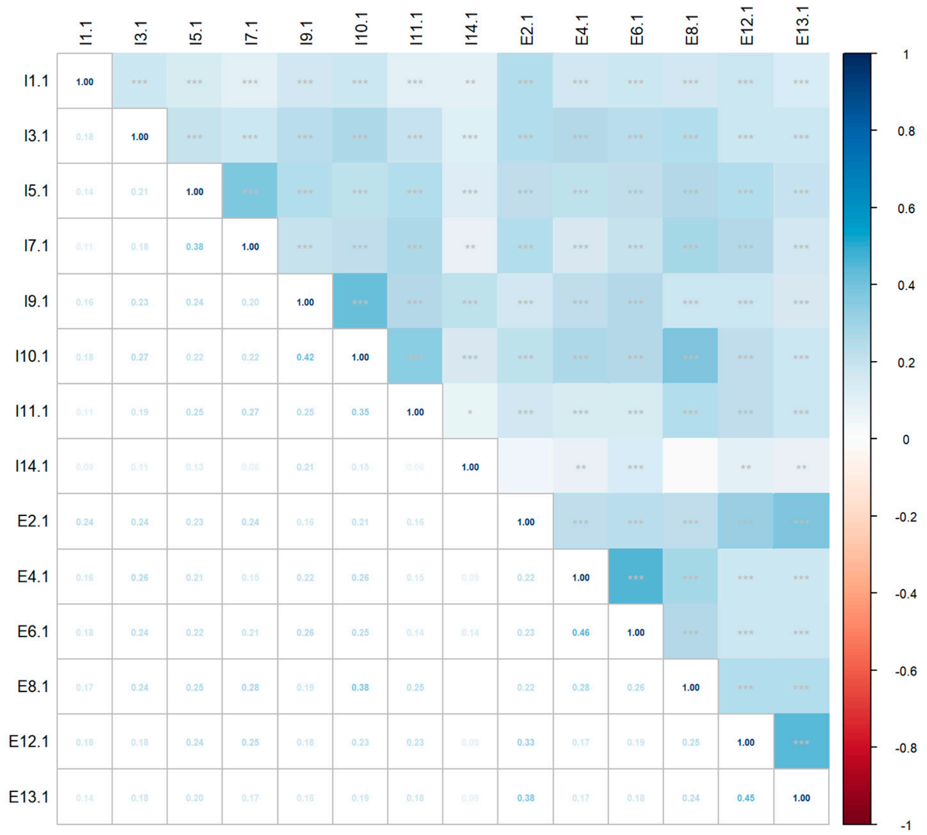

(b)

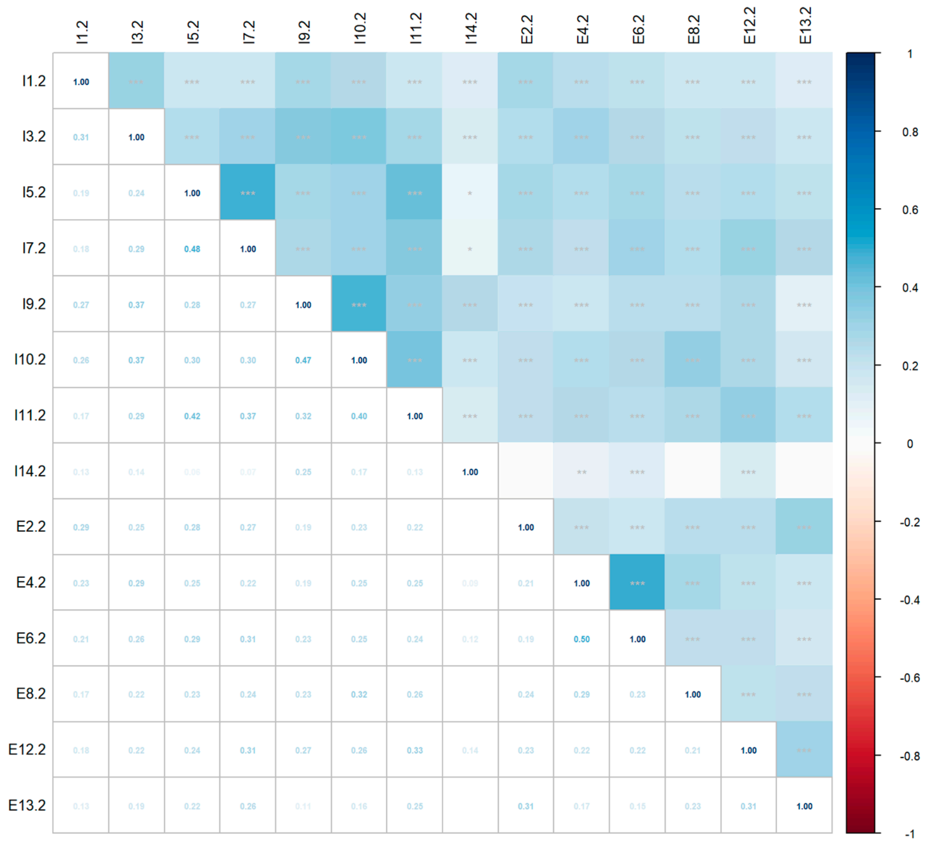

Figure S2. Correlation matrices of the 14 EBPs items at T1 and T2

Note. (a) Correlation between research variables in the T1 network model. (b) Correlation between research variables in the T2 network model. Node codes and corresponding item wording are provided in Table 2 of the main manuscript.

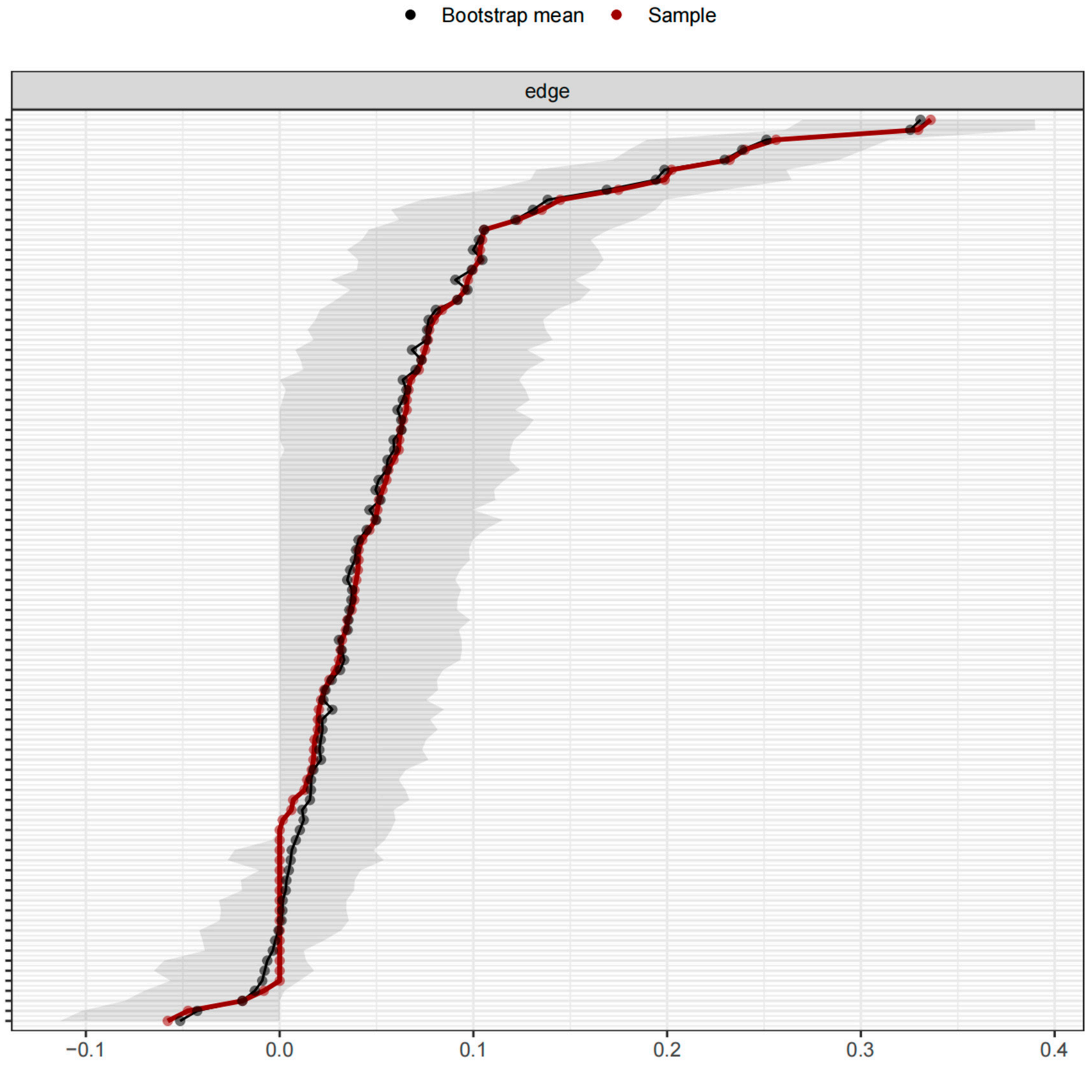

Figure S3. Bootstrapped 95% CI of estimated edge weights in T1

Note. Red points indicate the full-sample edge-weight estimates, black points indicate the bootstrap means, and gray intervals represent the 95% bootstrap confidence intervals.

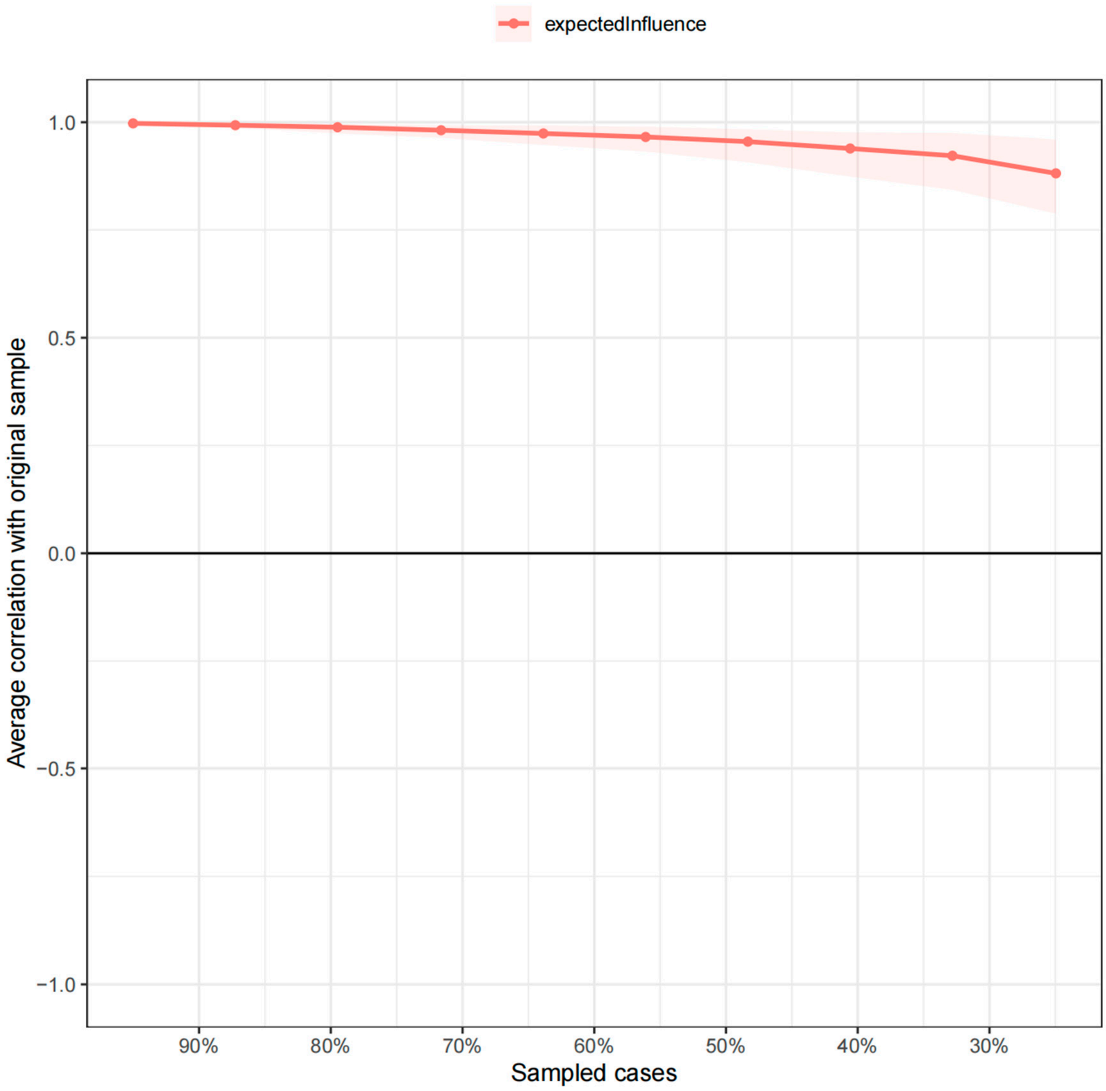

Figure S4. EI stability of the network in T1

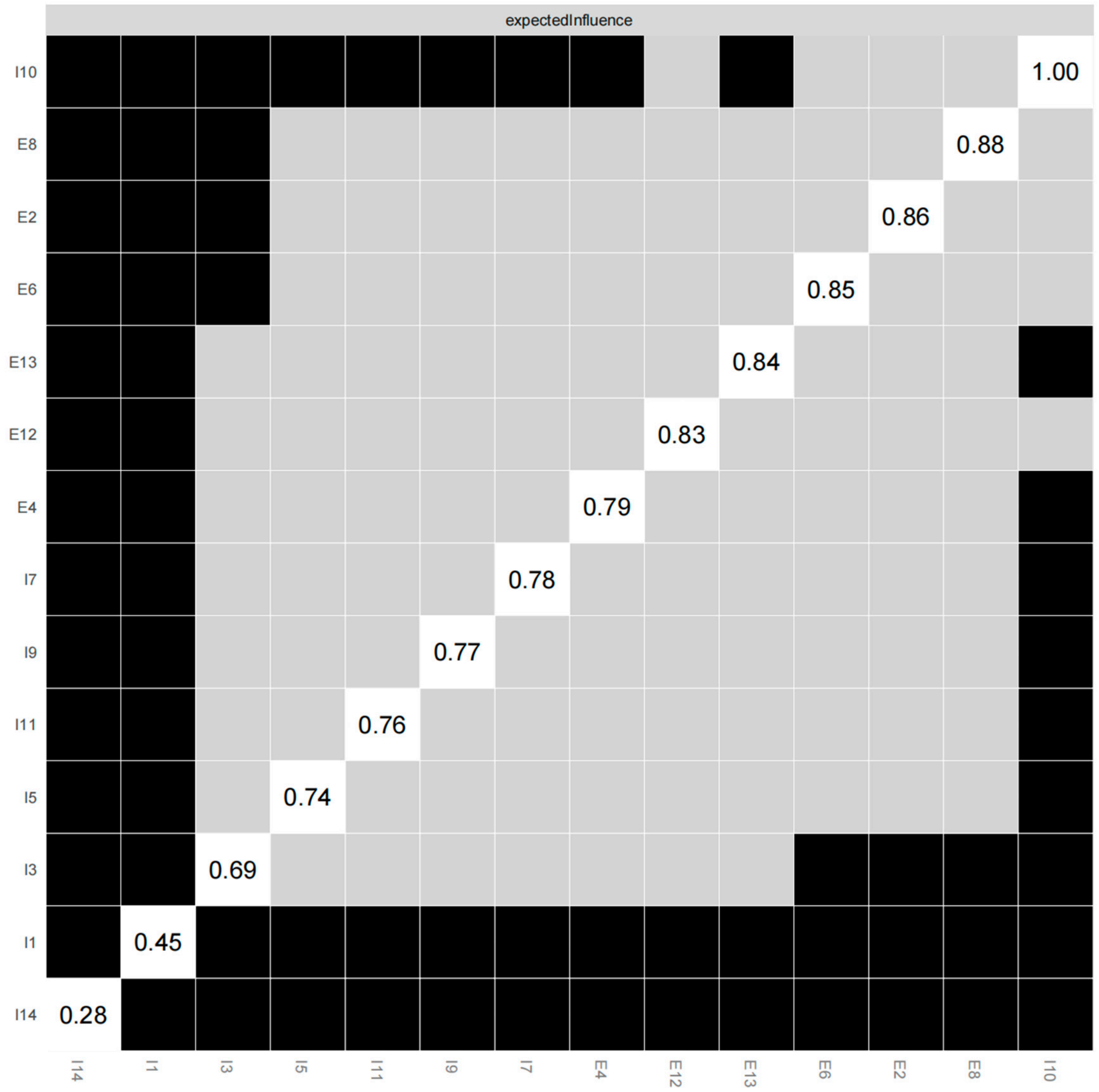

Figure S5. Bootstrapped test of node EI differences at T1

Note. Black cells indicate significant differences between node EI estimates at $\alpha = 0.05$, whereas gray cells indicate nonsignificant differences.

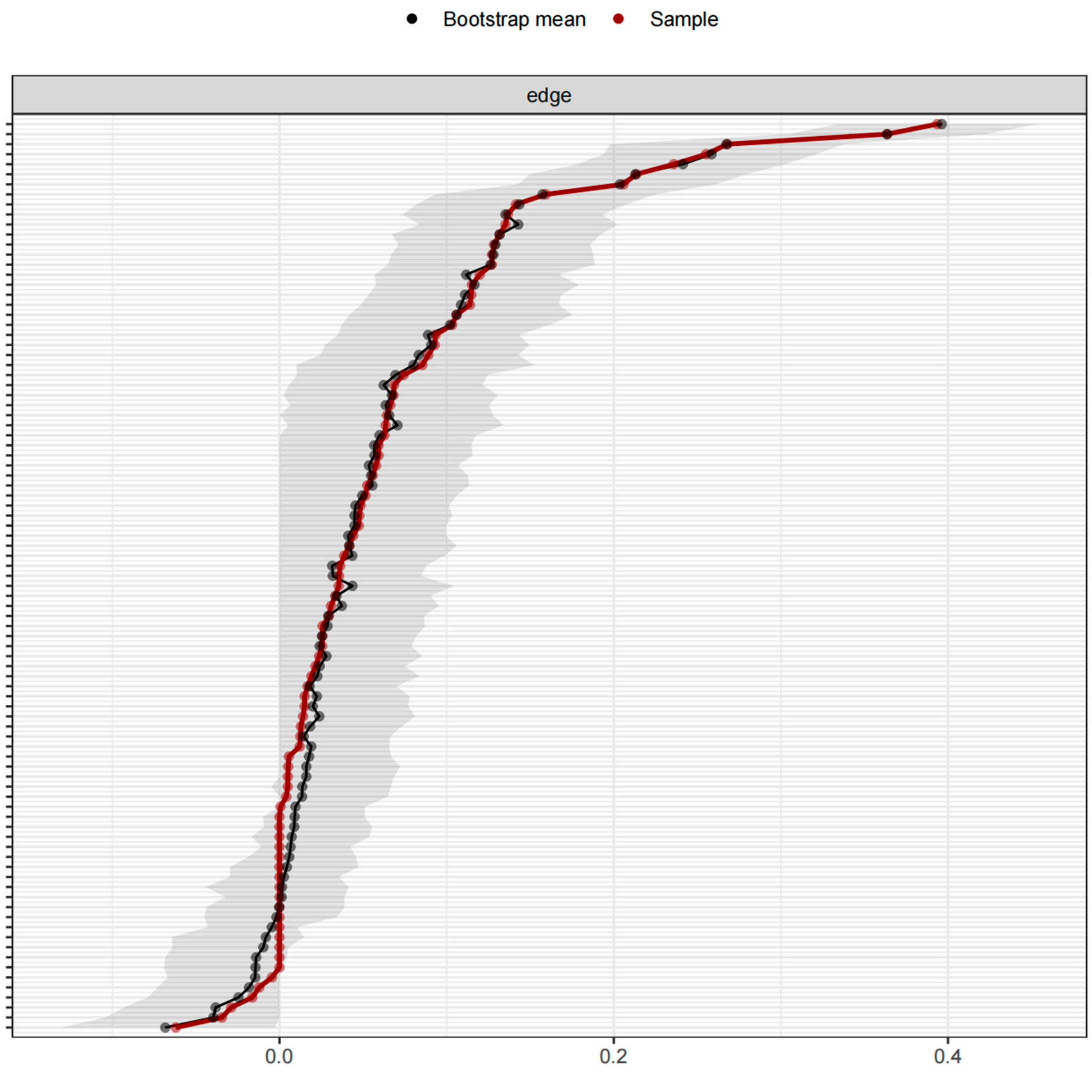

Figure S6. Bootstrapped 95% CI of estimated edge weights in T2

Note. Red points indicate the full-sample edge-weight estimates, black points indicate the bootstrap means, and gray intervals represent the 95% bootstrap confidence intervals.

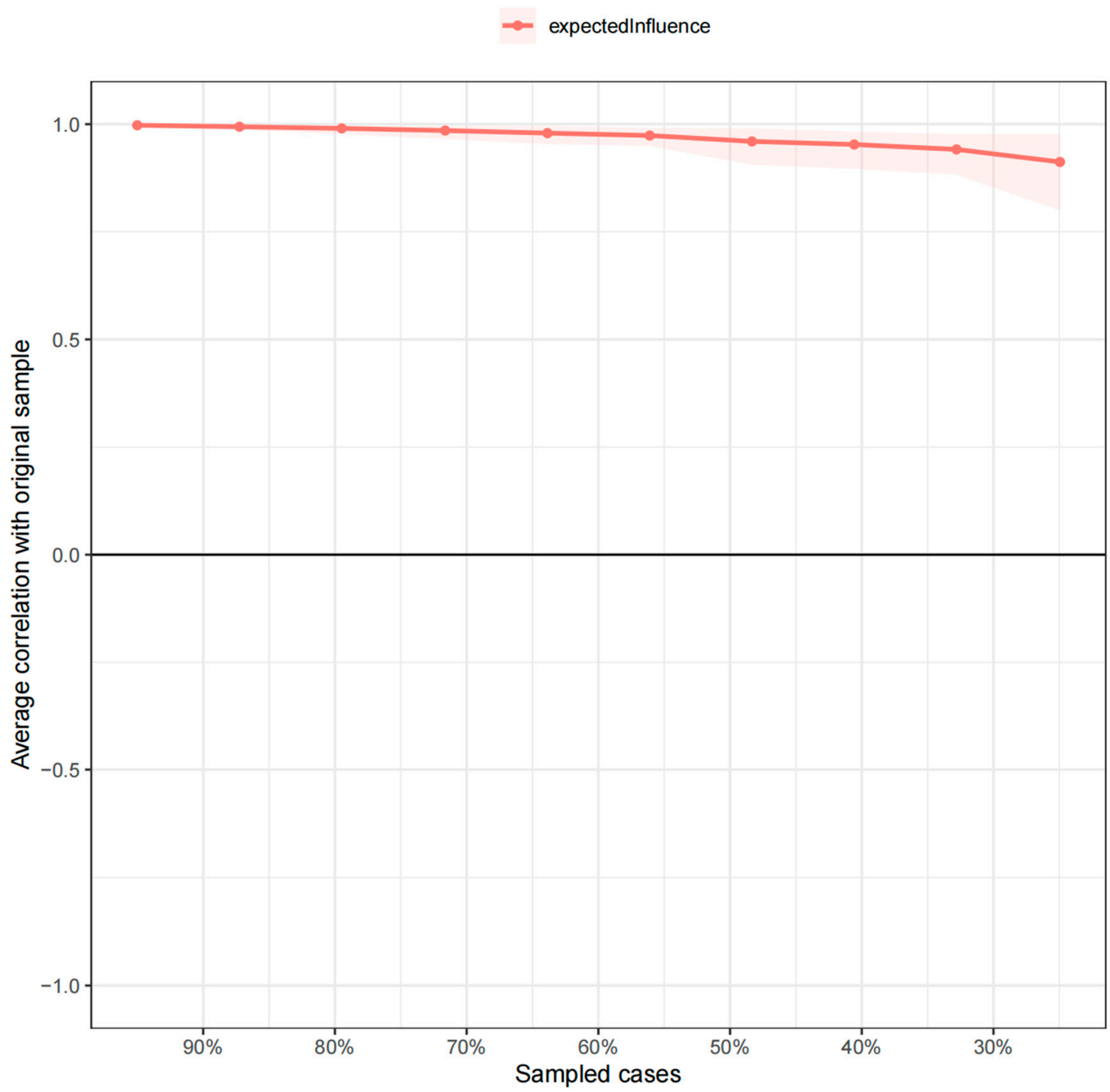

Figure S7. EI stability of the network in T2

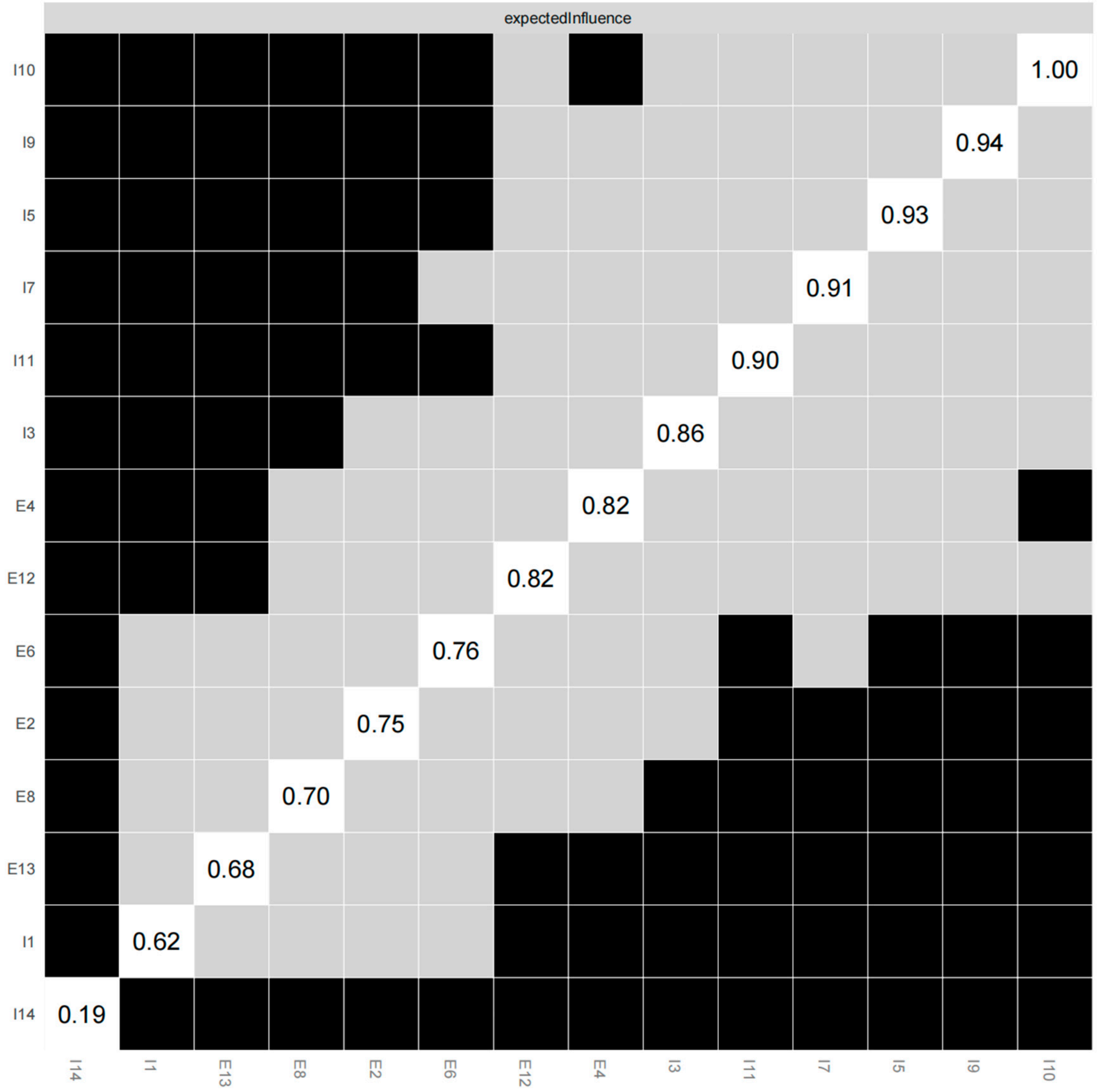

Figure S8. Bootstrapped test of node EI differences at T2

Note. Black cells indicate significant differences between node EI estimates at α = 0.05, whereas gray cells indicate nonsignificant differences.

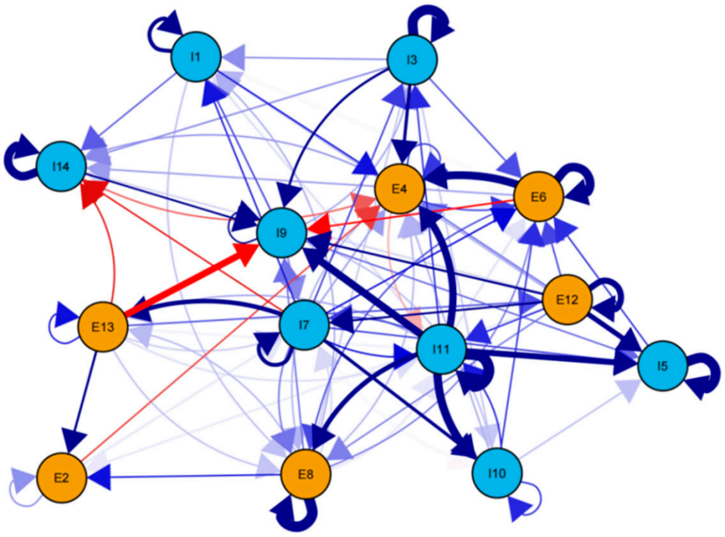

Internalizing problem behaviors

- I1: I feel angry when I have trouble learning
- I3: I worry about taking tests
- I5: I often feel lonely
- I7: I feel sad a lot of the time
- I9: I worry about doing well in school
- I10: I worry about finishing my homework
- I11: I worry about having someone to play with at school
- I14: I feel ashamed when I make mistakes at school

Externalizing problem behaviors

- E2: I often argue with other kids
- E4: It is hard for me to pay attention
- E6: I get distracted easily
- E8: It is hard for me to finish my school work
- E12: I get in trouble for talking and disturbing others
- E13: I get in trouble for fighting with other kids

Figure S9. Overall cross-lagged panel network including autoregressive paths

Note. Arrows indicate prospective associations from T1 to T2. Blue arrows indicate positive regression coefficients, whereas red arrows indicate negative regression coefficients. Arrow thickness represents the absolute magnitude of the Gaussian LASSO regression coefficient (*b*). Autoregressive paths are included.

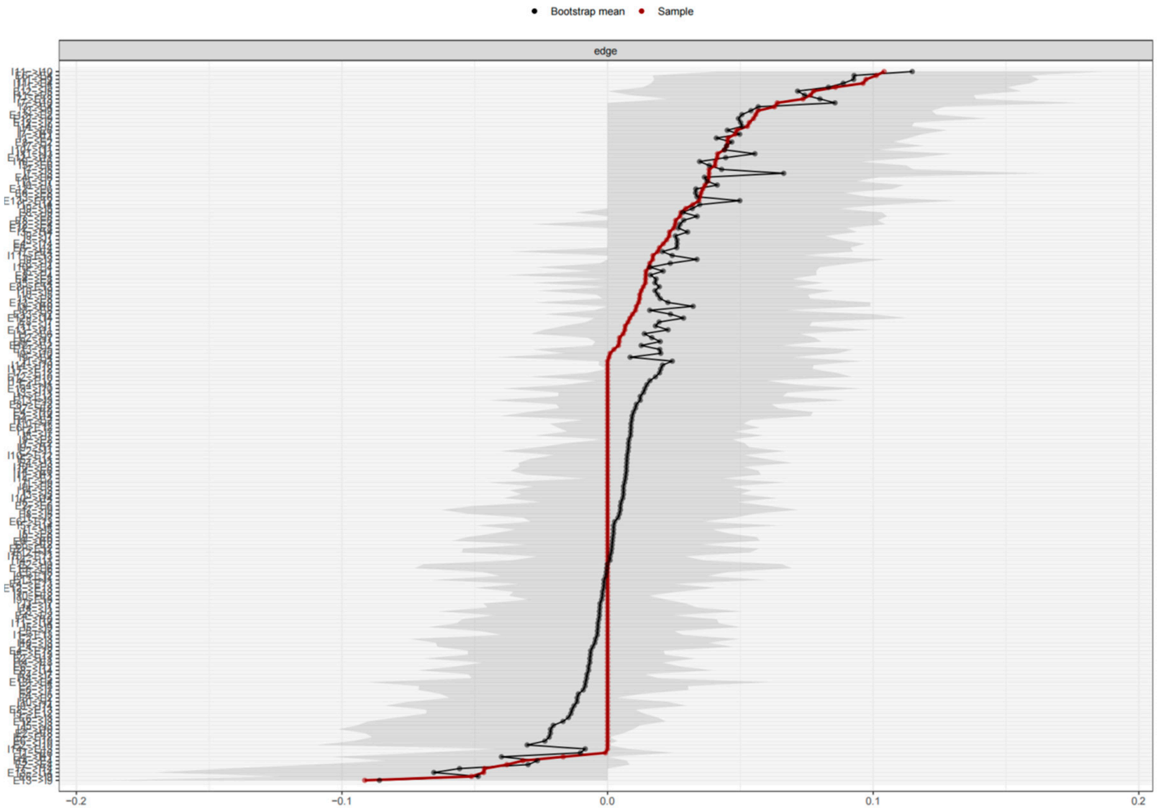

Figure S10. Bootstrap accuracy of edge-weight estimates in the overall cross-lagged panel network

Note. Red points are full-sample estimates, black points are bootstrap means, and gray intervals are 95% confidence intervals based on 1,000 bootstrap samples. Autoregressive paths were excluded.

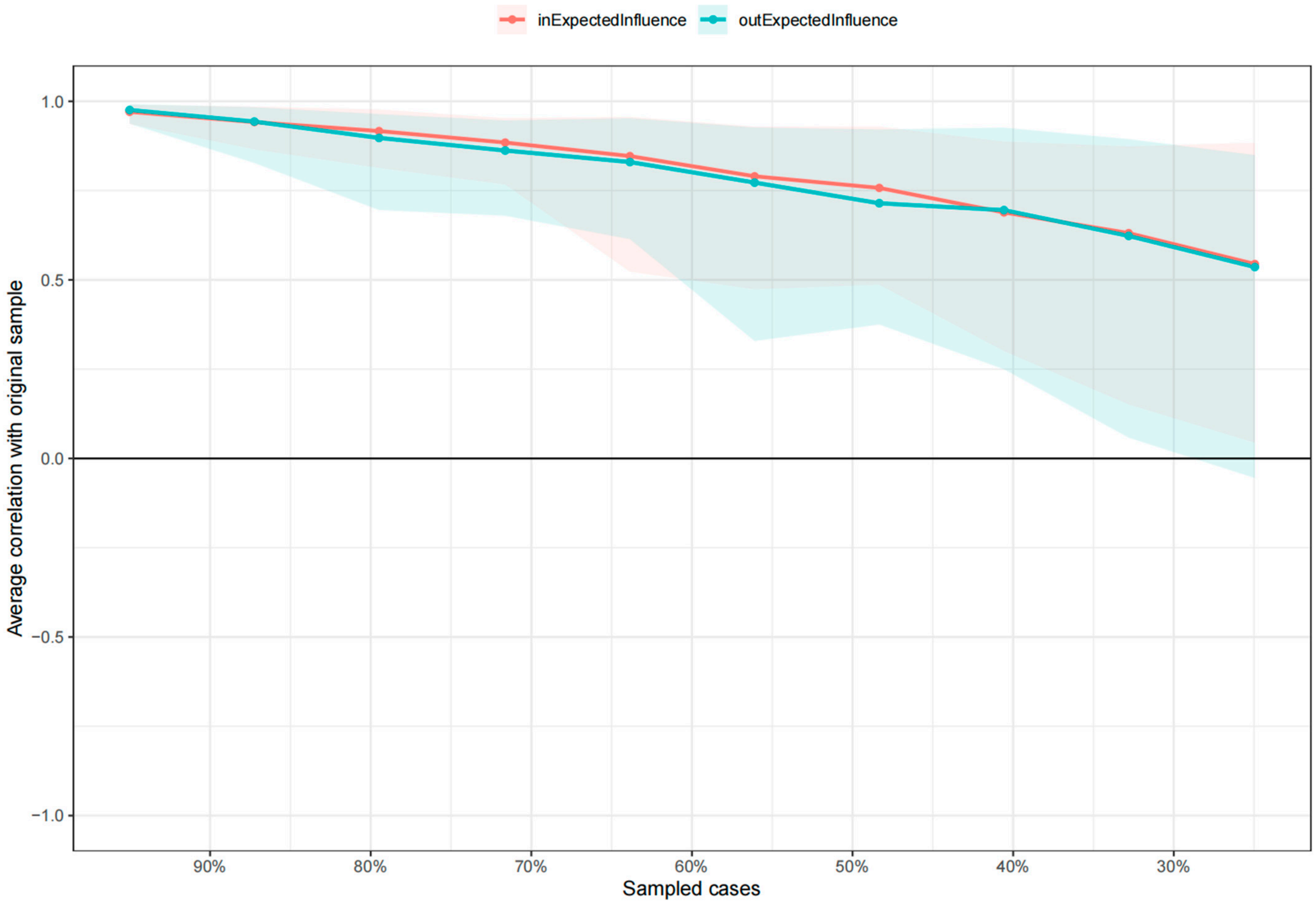

Figure S11. Case-dropping bootstrap stability of O-EI and I-EI in the overall cross-lagged panel network

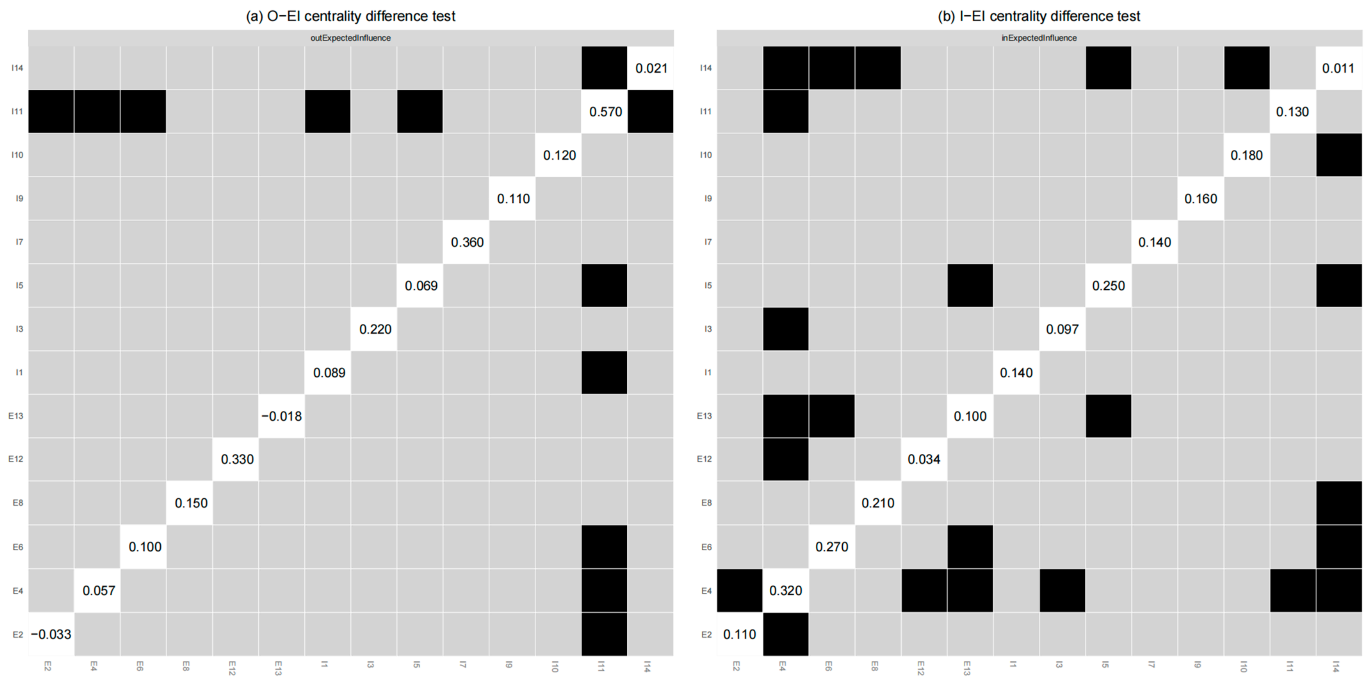

Figure S12. Bootstrap difference tests of O-EI and I-EI centrality in the overall cross-lagged panel network

Note. Panels (a) and (b) show pairwise bootstrap difference tests for O-EI and I-EI, respectively. Black and gray cells indicate significant and nonsignificant differences at α = 0.05. Diagonal values are full-sample centrality estimates. Autoregressive paths were excluded.

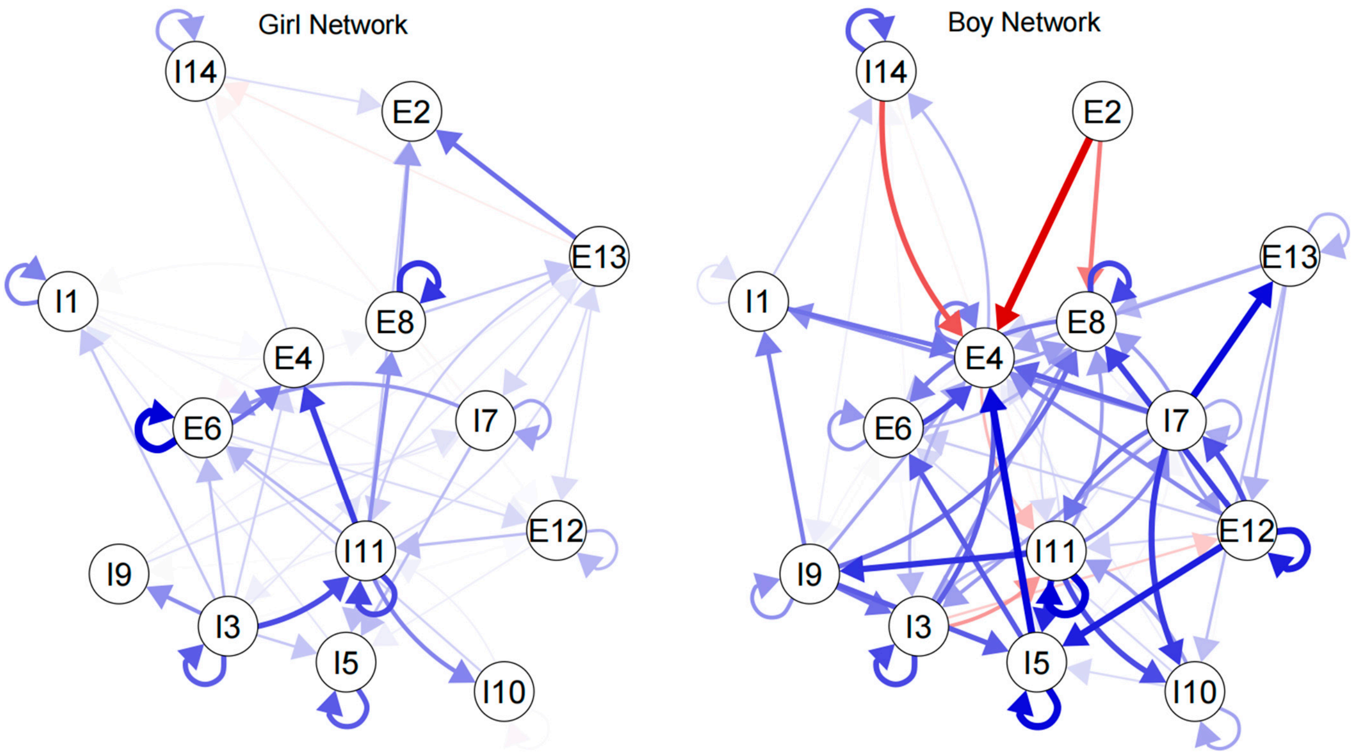

Figure S13. Cross-lagged panel networks for adolescent girls (left) and boys (right), including autoregressive and cross-lagged paths

Note. Arrows indicate prospective associations from T1 to T2. Blue arrows indicate positive regression coefficients, whereas red arrows indicate negative regression coefficients. Arrow thickness represents the absolute magnitude of the Gaussian LASSO regression coefficient (*b*). Autoregressive paths are included.

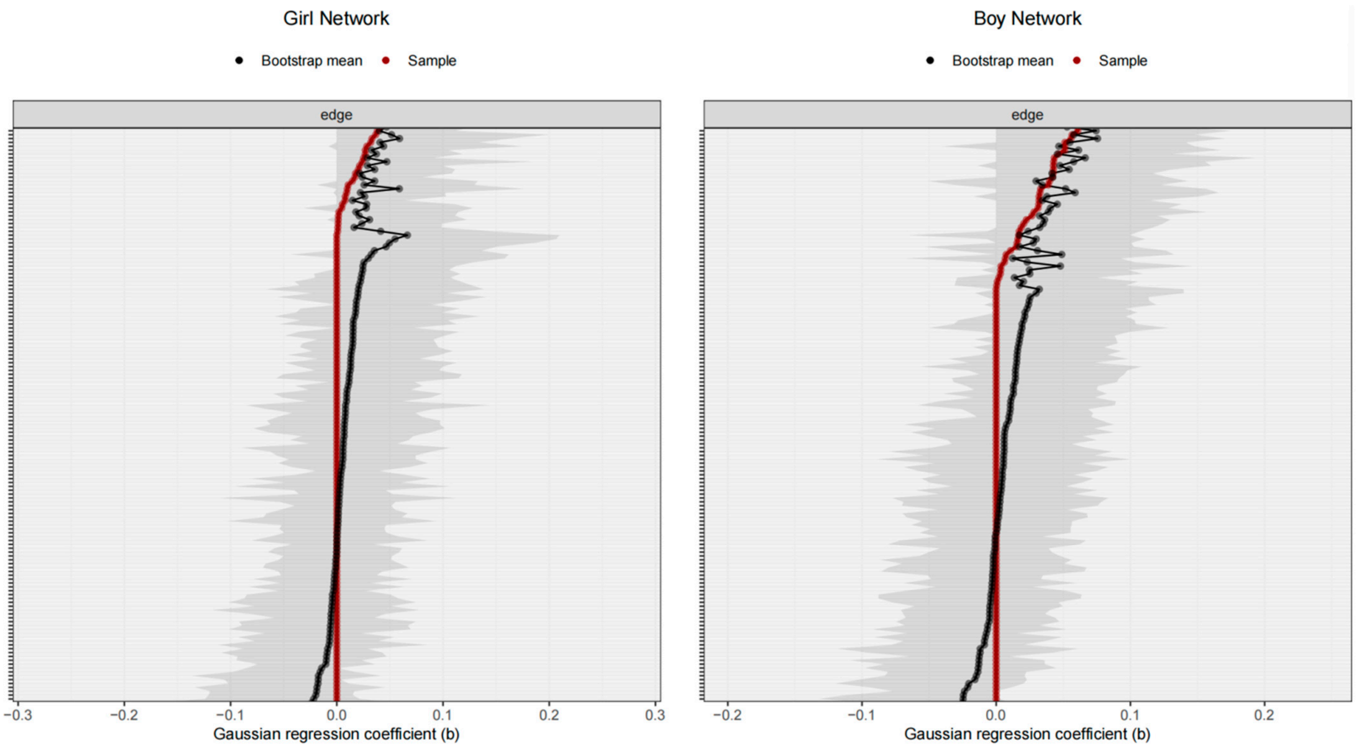

Figure S14. Bootstrap accuracy of edge-weight estimates in the gender-specific cross-lagged panel networks

Note. The left and right panels show the girls' and boys' networks, respectively. Red points are full-sample estimates, black points are bootstrap means, and gray intervals are 95% confidence intervals based on 1,000 bootstrap samples. Autoregressive paths were excluded.

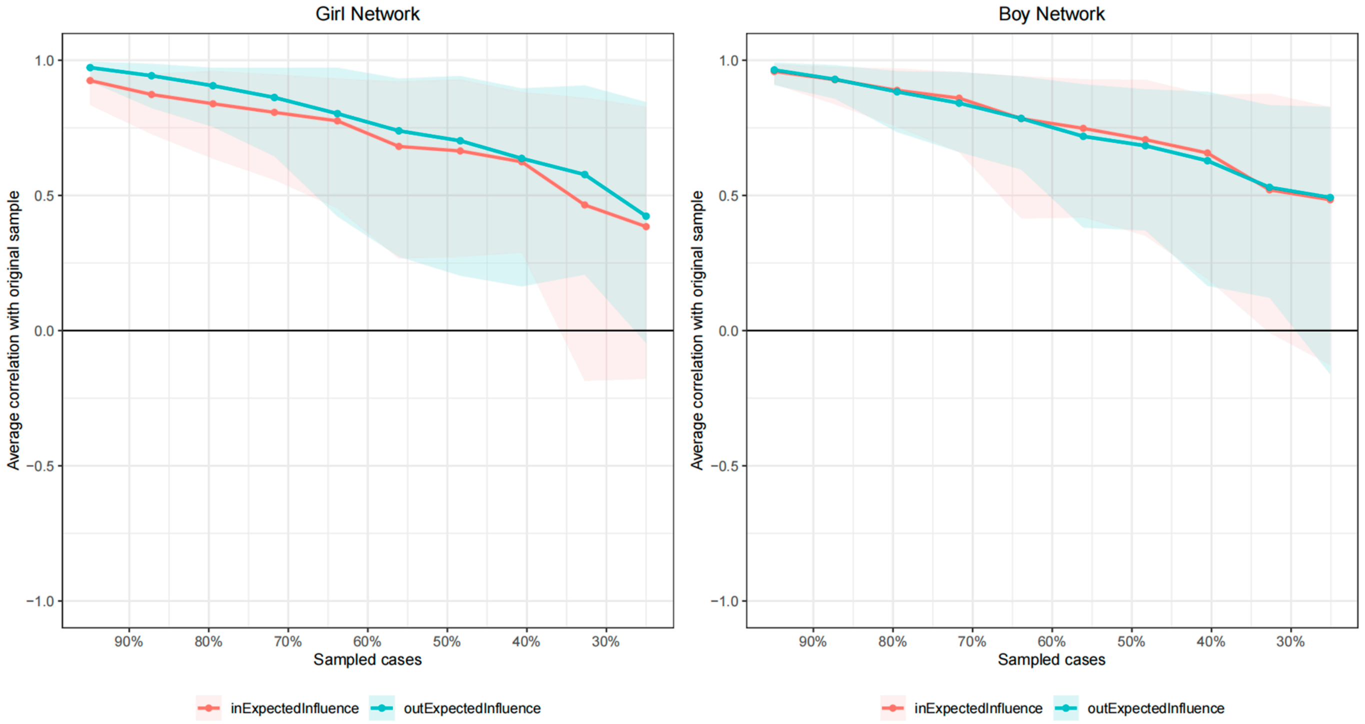

Figure S15. Case-dropping bootstrap stability of O-EI and I-EI in the gender-specific cross-lagged panel networks

Note. Stability was assessed using 1,000 case-dropping bootstrap samples. O-EI and I-EI were calculated from raw Gaussian regression coefficients (*b*), with autoregressive paths excluded.

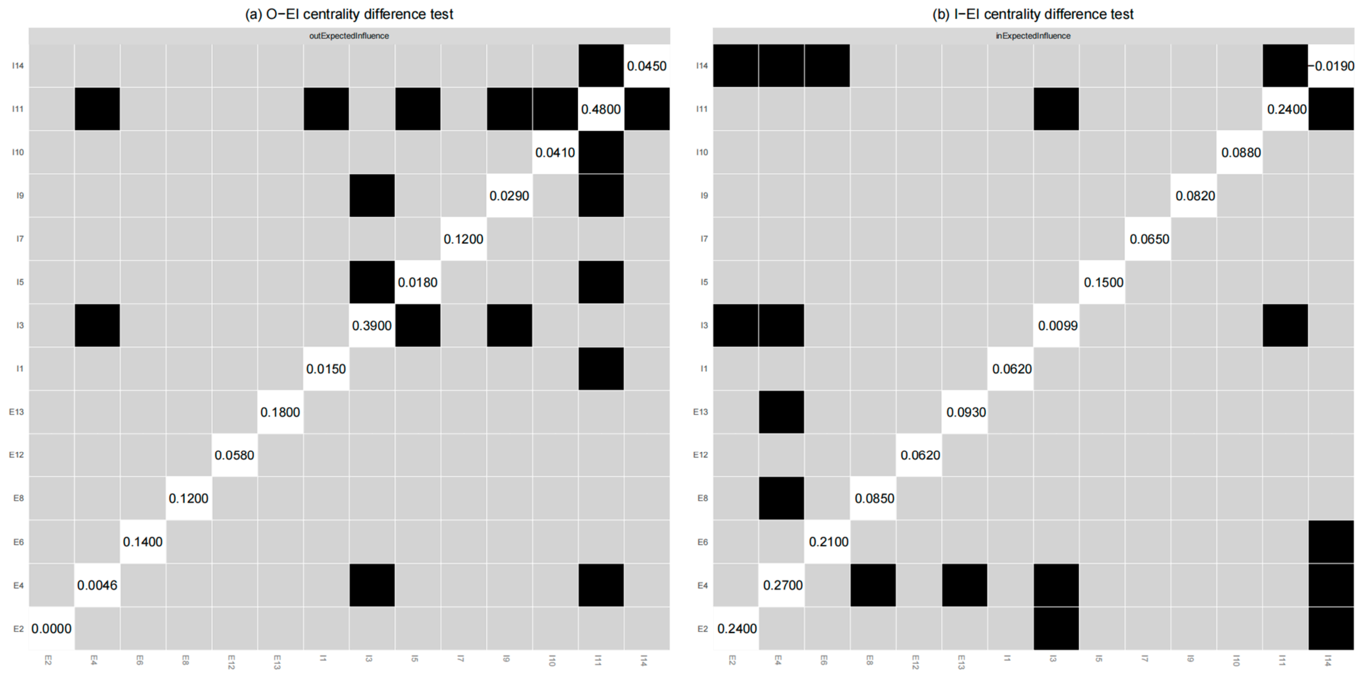

Figure S16. Bootstrap difference tests of O-EI and I-EI centrality in the girls' cross-lagged panel network

Note. Black and gray cells indicate significant and nonsignificant pairwise differences at α = 0.05, respectively. Diagonal values are full-sample centrality estimates. Autoregressive paths were excluded.

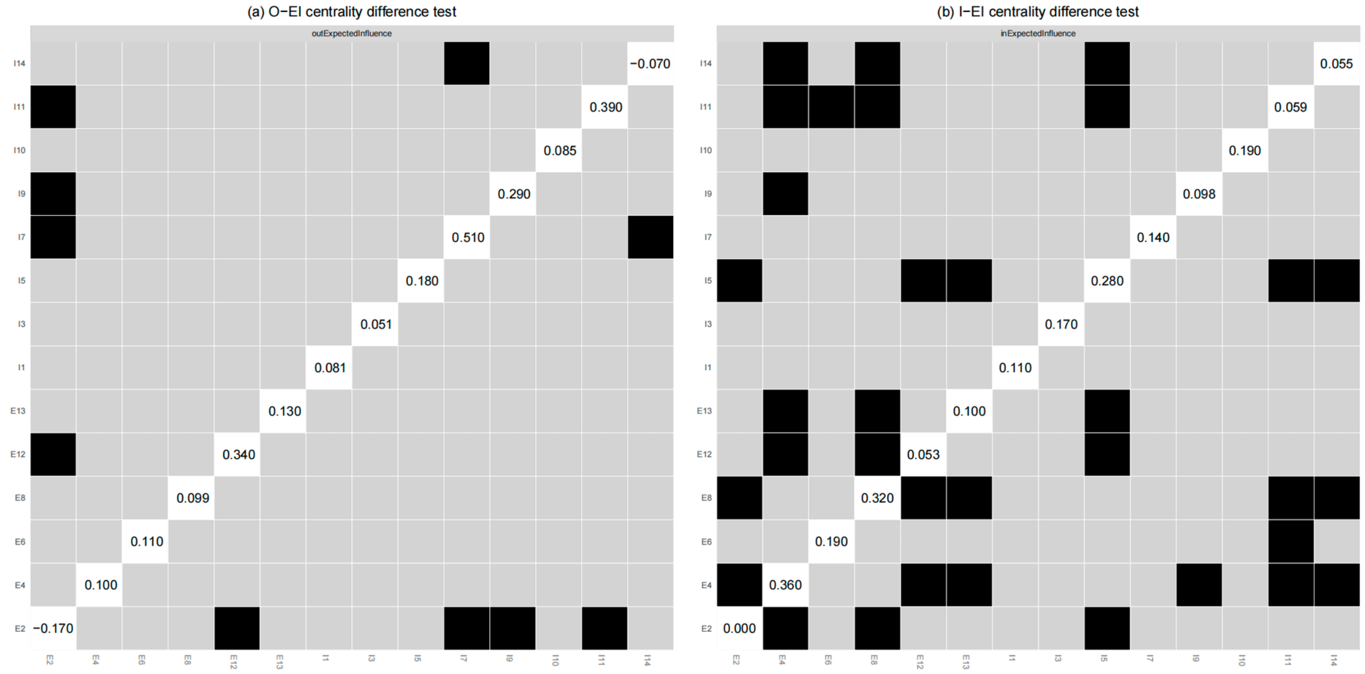

Figure S17. Bootstrap difference tests of O-EI and I-EI centrality in the boys' cross-lagged panel network

Note. Black and gray cells indicate significant and nonsignificant pairwise differences at α = 0.05, respectively. Diagonal values are full-sample centrality estimates. Autoregressive paths were excluded.

Table S1. Age distributions of the final analytic sample at T2 (*N* = 1,082)

Wave	Age, years	*n*	%
T2 (2022)	14	217	20.1
	15	217	20.0
	16	211	19.5
	17	199	18.4
	18	173	16.0
	19	65	6.0

Note. Percentages are based on the final analytic sample of 1,082 participants.

Table S2. Missing and invalid responses to EBPs items among participants successfully matched to T2 and meeting the age criteria at both waves (*N* = 1,513)

Wave	Items	Missing/invalid, *n* (%)
T1	All 14 EBPs items	50 (3.3%)
T2	I1, E2, I3, E4, I5, E6, I7, E8, I9, I10, I11, E12, and E13	296 (19.6%)
T2	I14	297 (19.6%)

Note. Percentages were based on *N* = 1,513. Missingness occurred predominantly at the questionnaire-block level: 50 participants had no valid T1 EBP responses and 296 had no valid T2 EBPs responses. One additional T2 response on I14 was invalid. Non-substantive or out-of-range responses were treated as missing/invalid.

Table S3. Baseline characteristics of participants included in and excluded from the final analytic sample

Baseline characteristic	Excluded participants	Included participants	*p*	Effect size
Age, years, *M* (*SD*)	12.66 (1.73), *n* = 1,525	12.09 (1.54), *n* = 1,082	< 0.001	*d* = 0.341
Male, *n* (%)	745 (52.8%), *n* = 1,411	590 (54.5%), *n* = 1,082	0.391	—
Agricultural, *n* (%)	1,087 (81.3%), *n* = 1,337	902 (83.4%), *n* = 1,082	0.187	*V* = 0.027
Internalizing problems, *M* (*SD*)	2.403 (0.665), *n* = 1,384	2.379 (0.642), *n* = 1,082	0.365	*d* = 0.037
Externalizing problems, *M* (*SD*)	2.124 (0.663), *n* = 1,382	2.104 (0.667), *n* = 1,082	0.468	*d* = 0.029

Note. Comparisons were based on available baseline data. Welch's *t* test was used for age, standard independent-samples *t* tests for internalizing and externalizing problems, and Pearson chi-square tests for gender and household registration status. Cohen's *d* and Cramér's *V* are reported as effect sizes.

Table S4. Node-redundancy assessment and CLPN sensitivity analyses for potentially overlapping items

Panel A. Goldbricker assessment

Item pair	Wave	Proportion of significantly different correlations	Inter-item correlation (r)	Suggested reduction
E4–E6	T1	0.083	0.454	No
E4–E6	T2	0.083	0.489	No
I10–E8	T1	0.250	0.379	No
I10–E8	T2	0.417	0.326	No

Panel B. CLPN sensitivity analyses under alternative item configurations

Model	N nodes	Matrix correlation with full-model retained-node submatrix	Top-10 edge overlap	Top three O-EI nodes	Top three I-EI nodes
Full 14-node model	14	—	—	I11, I7, E12	E4, E6, I5
E4 omitted	13	0.999	9/10	I11, I7, E12	I5, E6, E8
E6 omitted	13	0.996	10/10	I11, I7, E12	E4, I5, E8
I10 omitted	13	0.996	10/10	I11, E12, I7	E4, E6, I5
E8 omitted	13	0.999	10/10	I11, I7, E12	E4, E6, I5

Note. Goldbricker was applied to all 14 nodes at each wave using $p = 0.05$, a proportion threshold of 0.25, and a minimum inter-item correlation of 0.50; no node pair was suggested for reduction. Sensitivity analyses separately omitted E4, E6, I10, or E8. Matrix correlations and top-10 edge overlap were based on non-autoregressive cross-lagged coefficients. O-EI = out-expected influence; I-EI = in-expected influence.

Table S5. Preliminary domain-specific ordinal CFA model fit used to assess suitability for factorial measurement-invariance testing

Domain	Wave	χ^2	df	CFI	TLI	RMSEA [90% CI]	STDYX loading range
Internalizing (8 items)	T1	179.022	20	0.928	0.899	0.086 [0.074, 0.097]	0.257 – 0.684
Internalizing (8 items)	T2	315.731	20	0.932	0.905	0.117 [0.106, 0.128]	0.226 – 0.726
Externalizing (6 items)	T1	333.931	9	0.861	0.768	0.183 [0.166, 0.200]	0.545 – 0.731
Externalizing (6 items)	T2	273.261	9	0.885	0.808	0.165 [0.148, 0.182]	0.544 – 0.693

Note. N = 1,082. Separate one-factor ordinal CFA models were estimated for each domain at T1 and T2 using WLSMV. Because model fit was mixed to inadequate, hierarchical factorial invariance testing was not pursued.

Table S6. Item-level descriptive statistics and across-wave distributional differences at T1 and T2

Node	T1 *Mean* (*SD*)	T2 *Mean* (*SD*)	Max shift (pp)
I1	2.47 (1.11)	2.65 (1.19)	13.0
E2	1.92 (0.99)	1.73 (0.90)	7.9
I3	2.33 (1.20)	2.41 (1.28)	8.7
E4	2.59 (1.18)	2.61 (1.24)	10.2
I5	1.98 (1.12)	1.92 (1.05)	7.8
E6	2.67 (1.19)	2.72 (1.24)	12.7
I7	1.88 (1.02)	1.84 (0.98)	4.7
E8	1.92 (1.09)	1.89 (1.02)	9.9
I9	2.74 (1.22)	2.53 (1.27)	12.8
I10	2.32 (1.30)	2.32 (1.28)	5.6
I11	1.88 (1.15)	1.85 (1.08)	6.0
E12	1.90 (1.02)	1.89 (1.02)	5.9
E13	1.63 (0.93)	1.45 (0.78)	8.9
I14	3.45 (1.27)	3.49 (1.25)	11.2

Note. N = 1,082. pp = percentage points. Maximum category shift represents the largest absolute percentage-point difference across the five response categories between T1 and T2.

Table S7. Item-level descriptive statistics and gender-related distributional differences at T1 and T2

Node	Boys *Mean* (*SD*) (T1)	Girls *Mean* (*SD*) (T1)	Cramér's *V* (T1)	Boys *Mean* (*SD*) (T2)	Girls *Mean* (*SD*) (T2)	Cramér's *V* (T2)
I1	2.48 (1.14)	2.46 (1.07)	0.065	2.49 (1.17)	2.85 (1.19)	0.151
E2	1.93 (1.01)	1.89 (0.96)	0.046	1.77 (0.96)	1.70 (0.83)	0.070
I3	2.34 (1.21)	2.31 (1.18)	0.035	2.23 (1.26)	2.63 (1.26)	0.192
E4	2.64 (1.19)	2.54 (1.16)	0.053	2.52 (1.24)	2.71 (1.23)	0.101
I5	1.95 (1.11)	2.00 (1.12)	0.050	1.91 (1.07)	1.93 (1.02)	0.060
E6	2.77 (1.22)	2.55 (1.15)	0.101	2.63 (1.24)	2.83 (1.24)	0.109
I7	1.83 (0.98)	1.93 (1.06)	0.073	1.81 (1.01)	1.87 (0.95)	0.100
E8	2.00 (1.15)	1.82 (1.02)	0.095	1.93 (1.09)	1.84 (0.93)	0.106
I9	2.67 (1.22)	2.82 (1.22)	0.081	2.37 (1.23)	2.72 (1.29)	0.141
I10	2.32 (1.30)	2.31 (1.29)	0.034	2.18 (1.24)	2.49 (1.31)	0.125
I11	1.84 (1.15)	1.93 (1.14)	0.068	1.75 (1.02)	1.96 (1.14)	0.103
E12	1.97 (1.09)	1.81 (0.93)	0.098	1.93 (1.05)	1.85 (0.98)	0.044
E13	1.76 (1.00)	1.48 (0.80)	0.177	1.55 (0.84)	1.34 (0.69)	0.153
I14	3.42 (1.31)	3.48 (1.22)	0.109	3.49 (1.24)	3.48 (1.25)	0.038

Note. Boys n = 590; girls n = 492. Cramér's V was calculated from Pearson chi-square tests comparing the five-category response distributions between boys and girls at each wave and is reported as a descriptive measure of the magnitude of distributional differences.

Table S8. Numerical results of the overall cross-lagged panel network and bootstrap confidence intervals for focal cross-lagged paths

Panel A. Adjacency matrix of the overall cross-lagged panel network

T1 → T2	I1	I3	I5	I7	I9	I10	I11	I14	E2	E4	E6	E8	E12	E13
**I1**	—	0.000	0.000	0.000	0.000	0.000	0.000	0.032	0.000	0.045	-0.001	0.012	0.000	0.000
**I3**	0.022	—	0.000	0.000	0.057	0.011	0.000	0.023	0.000	0.063	0.035	0.011	0.000	0.000
**I5**	0.014	0.000	—	0.000	0.000	0.000	0.000	0.000	0.000	0.014	0.040	0.000	0.000	0.000
**I7**	0.045	0.038	0.038	—	0.000	0.064	0.048	-0.046	0.000	0.019	0.049	0.027	0.000	0.074
**I9**	0.037	0.017	0.029	0.023	—	0.001	0.000	0.000	0.000	0.001	0.000	0.000	0.000	0.000
**I10**	0.000	0.000	0.012	0.000	0.013	—	0.044	0.000	0.000	0.016	0.041	0.000	0.000	0.000
**I11**	0.007	0.041	0.086	0.038	**0.097**	**0.104**	—	0.000	0.000	0.096	0.006	0.076	0.000	0.017
**I14**	0.000	0.000	0.000	0.000	0.053	0.000	0.000	—	0.000	-0.032	0.000	0.000	0.000	0.000
**E2**	0.000	0.000	0.000	0.005	0.000	0.000	0.000	0.000	—	-0.038	0.000	0.000	0.000	0.000
**E4**	0.000	0.000	0.000	0.000	0.000	0.000	-0.017	0.021	0.000	—	0.038	0.014	0.000	0.000
**E6**	0.000	0.000	0.000	0.000	-0.051	0.000	0.000	0.020	0.000	**0.101**	—	0.035	0.000	0.000
**E8**	0.016	0.000	0.000	0.008	0.028	0.000	0.004	0.000	0.045	0.014	0.026	—	0.000	0.013
**E12**	0.000	0.000	0.078	0.053	0.055	0.000	0.041	0.008	0.004	0.025	0.036	0.025	—	0.000
**E13**	0.000	0.000	0.002	0.009	-0.091	0.000	0.006	-0.047	0.056	0.000	0.000	0.012	0.034	—

Panel B. Bootstrap confidence intervals for the 10 focal cross-lagged paths with the largest absolute full-sample coefficients

Path	*b*	Bootstrap mean	95% bootstrap *CI*
I11 → I10	0.104	0.115	[0.041, 0.186]
E6 → E4	0.101	0.093	[0.018, 0.159]
I11 → I9	0.097	0.093	[0.018, 0.162]
I11 → E4	0.096	0.089	[0.017, 0.158]
E13 → I9	-0.091	-0.086	[-0.188, 0.000]
I11 → I5	0.086	0.083	[0.015, 0.150]
E12 → I5	0.078	0.072	[0.000, 0.154]
I11 → E8	0.076	0.074	[0.011, 0.137]
I7 → E13	0.074	0.080	[0.023, 0.139]
I7 → I10	0.064	0.086	[0.000, 0.177]

Note. In Panel A, rows are T1 predictors and columns are T2 outcomes; entries are raw Gaussian LASSO regression coefficients (*b*), with autoregressive paths omitted. Panel B reports the 10 largest absolute cross-lagged coefficients and their 95% bootstrap confidence intervals based on 1,000 samples. Confidence intervals reflect estimation uncertainty and were not used to classify LASSO-retained paths as statistically significant.

Table S9. Numerical results of the gender-specific cross-lagged panel networks and bootstrap confidence intervals for focal cross-lagged paths

Panel A. Adjacency matrix of the girls' cross-lagged panel network

T1 → T2	I1	I3	I5	I7	I9	I10	I11	I14	E2	E4	E6	E8	E12	E13
**I1**	—	0.000	0.000	0.000	0.000	0.000	0.000	0.000	0.000	0.001	0.000	0.005	0.008	0.000
**I3**	0.042	—	0.040	0.000	0.081	0.000	0.127	0.000	0.000	0.033	0.049	0.000	0.001	0.017
**I5**	0.018	0.000	—	0.000	0.000	0.000	0.000	0.000	0.000	0.000	0.000	0.000	0.000	0.000
**I7**	0.000	0.000	0.047	—	0.000	0.000	0.000	-0.006	0.000	0.000	0.074	0.000	0.000	0.000
**I9**	0.000	0.000	0.000	0.022	—	0.000	0.000	0.000	0.000	0.000	0.000	0.000	0.000	0.008
**I10**	0.000	0.000	0.000	0.000	0.000	—	0.009	0.000	0.000	0.000	0.032	0.000	0.000	0.000
**I11**	0.001	0.010	0.047	0.000	0.001	0.088	—	0.000	0.038	**0.137**	0.052	0.080	0.000	0.027
**I14**	0.000	0.000	0.000	0.000	0.000	0.000	0.020	—	0.025	0.000	0.000	0.000	0.000	0.000
**E2**	0.000	0.000	0.000	0.000	0.000	0.000	0.000	0.000	—	0.000	0.000	0.000	0.000	0.000
**E4**	0.000	0.000	0.000	0.000	0.000	0.000	0.000	0.000	0.000	—	0.005	0.000	0.000	0.000
**E6**	0.000	0.000	0.000	0.015	0.000	0.000	0.000	0.000	0.000	0.102	—	0.000	0.027	0.000
**E8**	0.002	0.000	0.000	0.000	0.000	0.000	0.000	0.000	0.071	0.000	0.000	—	0.002	0.041
**E12**	0.000	0.000	0.011	0.000	0.000	0.000	0.047	0.000	0.000	0.000	0.000	0.000	—	0.000
**E13**	0.000	0.000	0.000	0.028	0.000	0.000	0.037	-0.013	0.103	0.000	0.000	0.000	0.024	—

Panel B. Adjacency matrix of the boys' cross-lagged panel network

T1 → T2	I1	I3	I5	I7	I9	I10	I11	I14	E2	E4	E6	E8	E12	E13
**I1**	—	0.000	0.000	0.000	0.000	0.000	0.000	0.021	0.000	0.061	0.000	0.000	0.000	0.000
**I3**	0.000	—	0.000	0.000	0.000	0.000	-0.045	0.003	0.000	0.069	0.001	0.046	-0.023	0.000
**I5**	0.000	0.000	—	0.003	0.000	0.000	0.000	0.000	0.000	**0.104**	0.071	0.000	0.000	0.000
**I7**	0.051	0.043	0.011	—	0.000	0.084	0.061	0.000	0.000	0.068	0.000	0.043	0.043	0.103
**I9**	0.056	0.063	0.070	0.000	—	0.000	0.000	0.000	0.000	0.039	0.000	0.063	0.000	0.000
**I10**	0.000	0.000	0.016	0.002	0.000	—	0.043	0.000	0.000	0.007	0.017	0.000	0.000	0.000
**I11**	0.000	0.032	0.092	0.051	0.087	0.076	—	0.000	0.000	0.015	0.000	0.042	0.000	0.000
**I14**	0.000	0.000	0.000	0.000	0.007	0.000	-0.004	—	0.000	-0.073	0.000	0.000	0.000	0.000
**E2**	0.000	0.000	0.000	0.000	0.000	0.000	0.000	0.000	—	**-0.110**	0.000	-0.057	0.000	0.000
**E4**	0.000	0.033	0.000	0.000	0.000	0.000	-0.030	0.031	0.000	—	0.027	0.042	0.000	0.000
**E6**	0.000	0.000	0.000	0.000	0.000	0.000	0.000	0.000	0.000	0.077	—	0.032	0.000	0.000
**E8**	0.000	0.000	0.000	0.006	0.004	0.000	0.016	0.000	0.000	0.017	0.057	—	0.000	0.000
**E12**	0.000	0.000	0.094	0.076	0.000	0.000	0.019	0.000	0.000	0.051	0.022	0.081	—	0.000
**E13**	0.000	0.000	0.000	0.000	0.000	0.028	0.000	0.000	0.000	0.040	0.000	0.032	0.033	—

Panel C. Bootstrap confidence intervals for the 10 focal cross-lagged paths with the largest absolute full-sample coefficients in girls

Path	*b*	Bootstrap mean	95% bootstrap *CI*
I11 → E4	0.137	0.164	[0.056, 0.277]
I3 → I11	0.127	0.134	[0.038, 0.227]
E13 → E2	0.103	0.099	[0.000, 0.247]
E6 → E4	0.102	0.119	[0.000, 0.228]
I11 → I10	0.088	0.131	[0.000, 0.247]
I3 → I9	0.081	0.103	[0.000, 0.215]
I11 → E8	0.080	0.095	[0.000, 0.181]
I7 → E6	0.074	0.086	[0.000, 0.210]
E8 → E2	0.071	0.069	[0.000, 0.165]
I11 → E6	0.052	0.063	[0.000, 0.174]

Panel D. Bootstrap confidence intervals for the 10 focal cross-lagged paths with the largest absolute full-sample coefficients in boys

Path	*b*	Bootstrap mean	95% bootstrap *CI*
E2 → E4	-0.110	-0.084	[-0.196, 0.000]
I5 → E4	0.104	0.089	[0.000, 0.185]
I7 → E13	0.103	0.149	[0.056, 0.242]
E12 → I5	0.094	0.099	[0.000, 0.199]
I11 → I5	0.092	0.095	[0.000, 0.192]
I11 → I9	0.087	0.115	[0.000, 0.213]
I7 → I10	0.084	0.085	[0.000, 0.212]
E12 → E8	0.081	0.077	[0.000, 0.171]
E6 → E4	0.077	0.072	[0.000, 0.164]
I11 → I10	0.076	0.072	[0.000, 0.173]

Note. Girls n = 492; boys n = 590. In Panels A and B, rows are T1 predictors and columns are T2 outcomes; entries are raw Gaussian LASSO coefficients (b), with autoregressive paths omitted. Panels C and D report the 10 largest absolute cross-lagged coefficients and their 95% bootstrap confidence intervals.

Table S10. Direct permutation-based comparisons of the girls' and boys' cross-lagged panel networks

Panel A. Global permutation tests of between-gender network differences

Test	Observed statistic	Permutation *p*
Maximum absolute edge difference	0.171	0.171
Global absolute connectivity difference	1.146	0.221

Panel B. Ten directed edges with the largest absolute observed girls-minus-boys differences

Path	Girls *b*	Boys *b*	Girls − Boys	|Difference|	Raw *p*	BH-FDR *p*	max-T FWER *p*
I3 → I11	0.127	-0.045	0.171	0.171	0.001	0.182	0.171
I11 → E4	0.137	0.015	0.122	0.122	0.098	0.856	0.857
E2 → E4	0.000	-0.110	0.110	0.110	0.013	0.788	0.962
I5 → E4	0.000	0.104	-0.104	0.104	0.005	0.455	0.984
E13 → E2	0.103	0.000	0.103	0.103	0.093	0.856	0.984
I7 → E13	0.000	0.103	-0.103	0.103	0.087	0.856	0.986
I11 → I9	0.001	0.087	-0.086	0.086	0.334	1.000	1.000
I7 → I10	0.000	0.084	-0.084	0.084	0.374	1.000	1.000
E12 → I5	0.011	0.094	-0.084	0.084	0.306	1.000	1.000
I3 → I9	0.081	0.000	0.081	0.081	0.156	0.997	1.000

Note. Differences are defined as girls minus boys. Panel A reports global permutation tests; Panel B reports individual directed-edge comparisons. Individual tests include raw, BH-FDR-adjusted, and max-T FWER-adjusted *p* values. No individual edge difference remained significant after multiple-comparison correction.
